# In vitro observation: the GFP-*E. coli* adhering to porcine erythrocytes can be removed by porcine alveolar macrophages

**DOI:** 10.7717/peerj.6439

**Published:** 2019-03-08

**Authors:** Wei Yin, Chun Wang, Kuohai Fan, Na Sun, Yaogui Sun, Hongquan Li

**Affiliations:** College of Animal Science and Veterinary Medicine, Shanxi Agricultral University, Taigu Shanxi, China

**Keywords:** Porcine erythrocytes, CR1-like, Porcine alveolar macrophages, GFP-*E. coli* removed

## Abstract

Although the activation of pathogen phagocytosis via complement system has been studied, erythrocyte-phagocyte interactions in pigs are not clearly understood. Therefore, we sought to investigate the ability of porcine erythrocytes to clear immune complexes (ICs) by using laser confocal microscopy and flow cytometry to observe the immune adhesion of porcine erythrocytes to fluorescent bacilli and the immune presentation process of transferring fluorescent bacilli to macrophages. Isolated porcine alveolar macrophages (PAMs) had uniform morphology and size, and a survival rate of 97.2%. The phagocytosis rate was 98.8%. After WT *E. coli* was labeled with Fluorescein Isothiocyanate (FITC), the bacteria showed a bright green fluorescence, and the labeling rate was 92.3%. When laser confocal microscopy was utilized to observe the co-incubation system of porcine erythrocytes, PAM, and fluorescent *E. coli*, the fluorescence intensity of bacilli decreased with increasing observation time and even disappeared. Flow Cytometry examination showed that the average fluorescence intensity of PAMs co-incubated with porcine erythrocytes adhered to WT-*E. coli*-FITC, was significantly higher than that of normal PAMs. Furthermore, when porcine erythrocytes adhered to WT *E. coli* were incubated with PAMs, the surface mean fluorescence intensity of porcine erythrocytes was significantly higher than that of the blank control group. This shows that PAMs can competitively bind to the oposinized *E. coli* adhered to the surface of porcine erythrocytes, and these oposinized pathogens can enter macrophages by the process of phagocytosis, which promoting the internalization of ICs or pathogens. During this process, the physical morphology of porcine erythrocytes was not damaged, but the levels of its main functional protein CR1-like were reduced.

## Introduction

Immune adherence is the most important immune function of human erythrocytes. The molecular basis and mechanism of the erythrocyte immune adherence have been well studied in biomedicine. The results confirmed that changes of erythrocyte immune adherence function are highly correlated with the occurrence, development, outcome, and immune status of the diseases (Khera & Das, 2009).

A large number of studies have shown that upon the activation of serum complement C3 by antigen or immune complex (IC) formed by the binding of antigen and specific antibody, C3 is cleaved into C3a and C3b, and C3b is deposited on the antigen or antigen/antibody complex to form C3b-antigen or C3b-antigen-antibody. The erythrocytes specifically bind to C3b by immune adherence and are transported to the reticuloendothelial system for the clearance of antigens and the inhibition of complement overactivation (Salam et al., 2018).

A variety of viruses, bacteria, tumor cells, etc. activate the human complement system and adhere to C3b, which are carried by ECR1 and rapidly transported to the reticuloendothelial phagocytic system for clearance (Lim et al., 2015; Ng et al., 1987). ECR1 adheres antigen or IC and is presented to phagocytes, promoting clearance of antigen or IC in the blood circulation. In human blood circulation, 95% of CR1 is present on the erythrocytes, and erythrocytes are 500–1,000 times more efficient in clearing C3b-antigen or C3b-antigen-antibody in the blood circulation than leukocytes. When certain congenital or acquired factors cause a decrease in the number or viability of the ECR1, IC would over-deposit in the tissue, causing serious tissue damage and various immune diseases such as rheumatoid arthritis and lupus erythematosus (Khera & Das, 2009). Therefore, the molecular mechanism of phagocytic clearance of IC mediated by the immune adherence of ECR1 is of great interests in the field of erythrocyte immune function research in recent years.

Veterinary studies have shown that the erythrocytes in non-primate mammals have immune adherence function (Zhu et al., 2011). Early studies in China have found changes in the function of erythrocyte immune adherence in animal disease. Various infectious diseases such as infectious bursal disease (Jiang et al., 2010), Marek’s disease (BAO ENDON SU JIANDONG, 2000), chicken inclusion body hepatitis (Feng & Cai, 2000) porcine cysticercosis (MA Hai li et al., 2003) are accompanied by a decrease in erythrocyte immune adherence function.

We observed that the porcine erythrocytes have immune adherence function (Sun et al., 2012). There are ECR1-like scattered on the membrane of the erythrocytes (Yin et al., 2015). Furthermore, we observed the distribution of porcine ECR1-like on the membrane and found that after the exogenous antigens were sensitized with fresh serum, the fluidity of the erythrocyte membrane increased, the ECR1-like changed from scattered distribution to cluster aggregation, and multivalent bond to C3b-IC, resulting in immune adherence (YinWei et al., 2015). However, to date, very few studies have investigated the process and mechanism of IC immune transport system of porcine erythrocytes. Therefore, the study on the molecular mechanism of IC transport system in the porcine erythrocytes is of great significance for a comprehensive understanding of the immune system and the immune network.

## Materials and Methods

### Animal ethics

All animals used in the present experiments were cared for humanely and the use of the animals was approved by the ethics committee at the Animal Science and Veterinary Medicine College of Shanxi Agriculture University in China (No. SXAU-ASVM-16019). All experiments were conducted in compliance with the International Guiding Principles for Biomedical Research Involving Animals (CIOMS and ICLAS, December 2012).

### Experimental animals and bacterial strains

Three Healthy Landrace pig, weighing 20 ± 2 kg, were purchased from Xinsihai pig farm in Wuxiang County, Shanxi Province, China. Healthy rabbits, weighing 2 ± 0.5 kg, were purchased from Shanxi Agricultural University Animal Production Laboratory. Wild type *Escherichia coli* (WT *E. coli*) cultured and then freeze-stored in our laboratory were used, The bacterial concentration measured by plate counting method was 4.846 × 105/mL.

### Main reagents and equipment

The main reagents include Indian ink (0.1%, Nanjing SenBeiJia Biological Technology Co., Ltd., Nanjing, China) mouse anti-porcine CR1-like monoclonal antibody (IgG2a; previously developed in our laboratory; FITC-goat anti-mouse IgG (Abbkine Inc., California, USA); mouse IgG1 isotype antibody (Beijing Protein Innovation Co. Ltd., Beijing, China); penicillin, streptomycin, Hank’s Buffer, phosphate-buffered saline (PBS, pH = 7.0), and Trypan blue solution (Solarbio Life Sciences Co. Ltd., Beijing, China; FITC and DMSO Sigma Inc., Missouri, USA); tryptone and yeast extract (Sangon Biotech Co. Ltd., Shanghai, China); erythrocyte lysis buffer (Tiangen Biotech Co. Ltd., Beijing, China); and BSA (Jackson ImmunoResearch Inc., rural Pennsylvania, USA). The main equipment used included: BD FACScalibur flow cytometer (BD Bioscience, New York, USA), and IX81 inverted fluorescence microscope and FV1000 laser confocal microscope (Olympus Corporation, Shinjuku, Tokyo, Japan).

### Preparation of porcine erythrocyte suspension

Blood was collected aseptically from the anterior vena cava from the experimental pigs, and lymphocyte separation medium was used to isolate porcine erythrocytes. These erythrocytes were resuspended using 0.5% IgG-free BSA-Hank’s buffer and were observed and counted under a normal light microscope. The cell density was adjusted to 2.47 × 10^7^/mL for use in further experiments.

### Isolation of porcine alveolar macrophages and primary culture

Pigs were anesthetized with ketamine and their lungs were obtained by aseptic surgery, Phosphate buffer saline (PBS) was used to flush the lung surface to remove contaminants. Then, 100 mL PBS (pH 7.4) was aspirated and injected into the lungs through the trachea, and the lavage fluid was collected and centrifuged at 112*g* for 5 min. The supernatant was discarded, and the cells were resuspended in RPMI 1,640 medium. This procedure was repeated twice to obtain porcine alveolar macrophages (PAMs). The PAM suspension was pipetted into a 1.5 mL centrifuge Tube and centrifuged at 112*g* for 5 min. The supernatant was discarded and the cells were resuspended in 12 mL of 1,640 cell culture medium. The cells were then seeded in six-well plates at a density of 2 × 106 cells/well. After incubation in a cell culture incubator for 12 h, the plates were removed and the culture medium was discarded. PBS was used to gently wash the plates and fresh 1,640 cell culture media was added at two mL/well. The plates were subsequently placed in a cell culture incubator and the cell culture medium was changed once every 24 h.

### PAM survival rate and phagocytosis rate

Porcine alveolar macrophage suspension (100 μL) was mixed with an equal volume of 0.4% Trypan blue. After staining for 2–3 min, the stained and unstained cells were counted to calculate the survival rate. The PAM suspension was pipetted and seeded at a density of 1 × 10^5^/mL in 24-well plates. After culturing for 2 h in a cell culture incubator, 100 μL of ink (autoclaved, diluted 10× in PBS) was added to each well while an equal volume of PBS was added to the control group. Each experiment was performed in triplicates.

### FITC labeling of WT *E. coli*

FITC powder (two mg) was collected in the dark and dissolved in 10 mL of PBS (pH 8.4). Then, 500 μL of this FITC solution was added to one mL of WT *E. coli* suspension. The mixture was incubated in a shaking incubator at 37 °C, 220 r/min for 1 h in the dark. After incubation, the solution was centrifuged at 2,795 g for 5 min, and the supernatant was discarded. The bacterial pellet was resuspended in one mL of 0.5% BSA-Hank’s buffer; this step was repeated twice. The fluorescence-quenching rate of the resuspended fluorescent bacteria was calculated at time points of 1 h, 1.5 h, 2 h, 2 h 5 min, 2 h 10 min, 2 h 15 min, 2 h 30 min, 2 h 45 min, and 3 h.

### Preparation of oposinized *E. coli*

Blood (two mL) was collected from the hearts of the experimental rabbits in ordinary blood collection tubes with no anticoagulant. These tubes were placed in a water bath, maintained at 37 °C, for 30 min and then centrifuged at 2,795*g* for 15 min. The serum thus obtained was collected for further use. Then, 50 μL of rabbit serum was added to 200 μL FITC-labeled WT *E. coli* suspension. The mixture was incubated in the dark in a water bath at 37 °C for 30 min and centrifuged at 2,795*g* for 5 min. The supernatant was discarded, and 200 μL of 0.5% BSA-Hank’s solution was used to resuspend the pellet. This procedure was repeated twice and the bacteria were observed under the microscope for any abnormalities before use.

### Detection of competitive binding of PAM to oposinized WT *E. coli*

A total of 200 μL of porcine erythrocyte suspension and 200 μL of the oposinized *E. coli*-FITC suspension were mixed and incubated at 37 °C in the dark for 30 min. The mixture was then centrifuged, and the pellet was washed twice and resuspended. Two milliliters of PAM solution was added to sterile six-well plates and incubated for 15 min. Then, the prepared cell suspension was added and the plates were incubated at 37 °C for 2 h. The cells were then harvested and 600 μL of erythrocyte lysis buffer was added. The suspension was centrifuged and washed twice. The pellet was resuspended in 300 μL of 1% paraformaldehyde fixing solution. The average fluorescence intensity of PAM was measured by flow cytometry. Statistical analysis of average fluorescence intensity of samples was carried out.

### Detection of CR1-like on porcine erythrocytes during transfer of oposinized *E. coli*

The oposinized *E. coli* suspension, porcine erythrocyte suspension, and PAM suspension was prepared as described in the previous sections. Five sterile 1.5 mL centrifuge Tubes were labeled as groups I, II, III, IV, and V. In groups I–IV, 100 μL of porcine erythrocyte suspension was added. In group I, 100 μL of oposinized *E. coli* suspension was added, incubated for 30 min, centrifuged, and washed twice. The bacteria were then resuspended in 100 μL of PAM suspension and mixed evenly. In group II, only 100 μL of PAM suspension was added and mixed evenly. Groups I and II were incubated for 2 h; erythrocytes were collected and sequentially incubated in the dark at 37 °C with mouse anti-porcine CR1-like monoclonal antibody and FITC-goat anti mouse IgG2. Groups III–V were control groups for flow cytometry: Group III had anti-porcine CR1-like monoclonal antibody, and FITC-goat anti mouse IgG2 added sequentially as described earlier. In group IV, mouse isotype control antibodies were added at an antibody dilution of 1:200, according to the instruction manual. In group V, no cells were added, but PBS was added as a control. The above groups were loaded in flow cytometry tubes and sent to Shanxi Medical University for quantitation of the average fluorescence intensity of erythrocytes (Table 1).

**Table 1:** The treatment of groups.

Treatment\Group	I (μL)	II (μL)	III (μL)	IV (μL)	V (μL)
Porcine erythrocyte	100	100	100	100	
PAM	100	100			
Oposinized *E. coli*	100				
CR1-like monoclonal antibody IgG2	10	10	10	10	
FITC-goat anti mouse antibodies	0.5	0.5	0.5		
Isotype control				0.5	
PBS	\				300

## Results

### Ink phagocytosis test result

**Figure 1:**
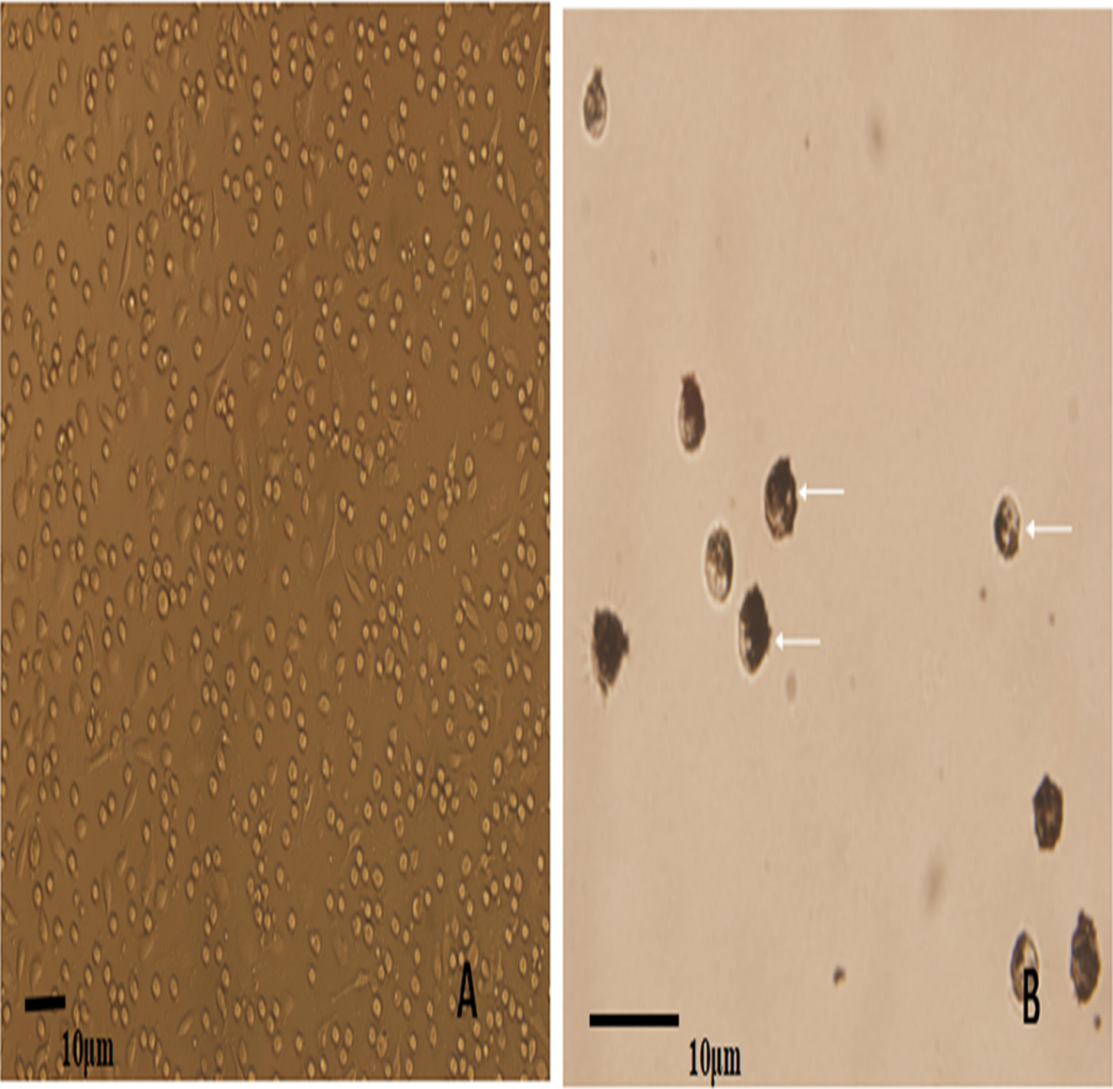
The microscopic examination for the recovery and phagocytic activity of PAM. (A) shows a representative image of the PAM culture morphology; (B) shows a representative image of the PAM phagocytized ink particles (as indicated by the arrow).

Porcine alveolar macrophages obtained after isolation were allowed to revive and were cultured. Inverted microscopy observations showed that most of the cells were circular, morphologically uniform, the PAM have phagocytic function, and were in a semi-adherent state. The survival rate of cells calculated after Trypan blue staining was 97.2% (Fig. 1A). Ink was added to the PAM culture system for continuing culture. Inverted microscopy observations of the ink-stained cells showed the phagocytosed particles inside the cells, and the PAM phagocytosis rate was calculated to be 98.8% (Fig. 1B).

### WT *E. coli* were successfully labeled with FITC

**Figure 2:**
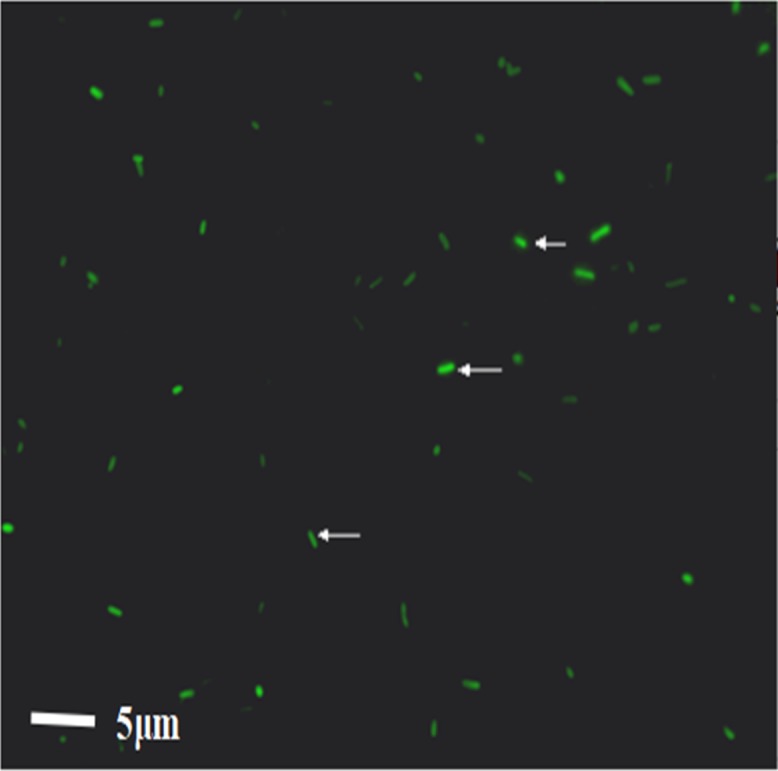
The result of the WT-*E. coli* labeled by FITC.

After WT *E. coli* were labeled with FITC dye, they were placed under a direct microscope. The bacteria appeared as short rods with size of one to two μm. Under the fluorescence view, WT *E. coli* presented a strong green fluorescence, demonstrating the successful labeling of bacteria; 92.3% bacteria were fluorescently labeled (Fig. 2). The bacteria were resuspended in PBS buffer and transferred to a laser confocal microscope to observe the fluorescence quenching time. Under a fluorescence field, the green fluorescence of the bacteria was gradually quenched from 0 to 3.4 h. The quenching rates were 3.7%, 10.9%, 11.2%, 11.7%, 14.6%, and 41.9%, at 1.5 h, 2 h, 2 h 15 min, 2 h 30 min, 3 h, and 3 h 45 min, respectively.

### WT-*E. coli*-FITC were bound and internalized by PAMs

**Figure 3:**
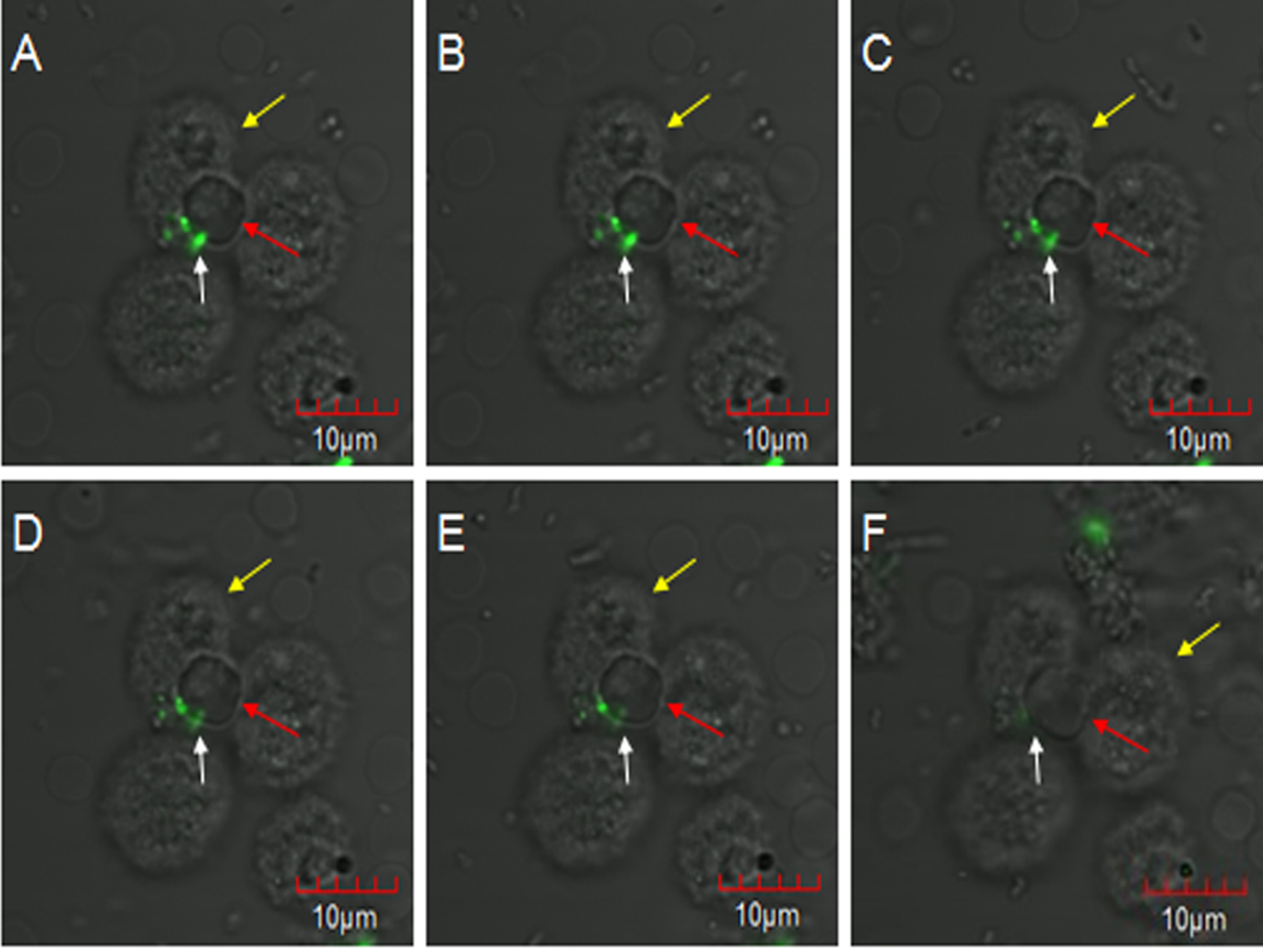
The observation of PAM competive binding reaction. At 2 h 5 min, 7 o’clock direction on a porcine erythrocyte had three fluorescent bacteria attached to it, and this erythrocyte was located at the junction of three PAMs (A). At 2 h 11 min, the fluorescence at 11 o’clock direction became slightly weaker (B). At 2 h 23 min, 2 h 27 min, and 2 h 36 min, bacterial fluorescence intensity weakened gradually (C–E). At 2 h 59 min, no fluorescent bacteria were seen at the 7 o’clock direction on the porcine erythrocyte (F).

*E. coli* oposinized by fresh rabbit serum was first incubated with erythrocytes and then co-incubated with PAMs, and subjected to laser confocal microscopy to observe the transfer process. The fluorescent bacteria were attached to the surface of porcine erythrocytes or PAMs, and there were no considerable changes in the adhesion status of fluorescent bacteria from 0 to 2 h. At 2 h 5 min, 7 o’clock direction on a porcine erythrocyte had three fluorescent bacteria attached to it, and this erythrocyte was located at the junction of three PAMs (Fig. 3A). At 2 h 11 min, the fluorescence at 11 o’clock direction became slightly weaker and the fluorescence became clearer after focusing. However, the contours of porcine erythrocytes showed a blurring phenomenon (Fig. 3B). At 2 h 23 min, 2 h 27 min, and 2 h 36 min, bacterial fluorescence intensity weakened gradually (Figs. 3C–3E). At 2 h 59 min, no fluorescent bacteria were seen at the 7 o’clock direction on the porcine erythrocyte. Thus, as the observation time increases, the weakening of the fluorescence intensity of the bacteria is not due to the quenching of fluorescence but is caused by the movement of fluorescent bacteria from their original adhesion location to the junction between erythrocytes and PAMs.

### Competitive binding of PAM to fluorescent *E. coli*

**Figure 4:**
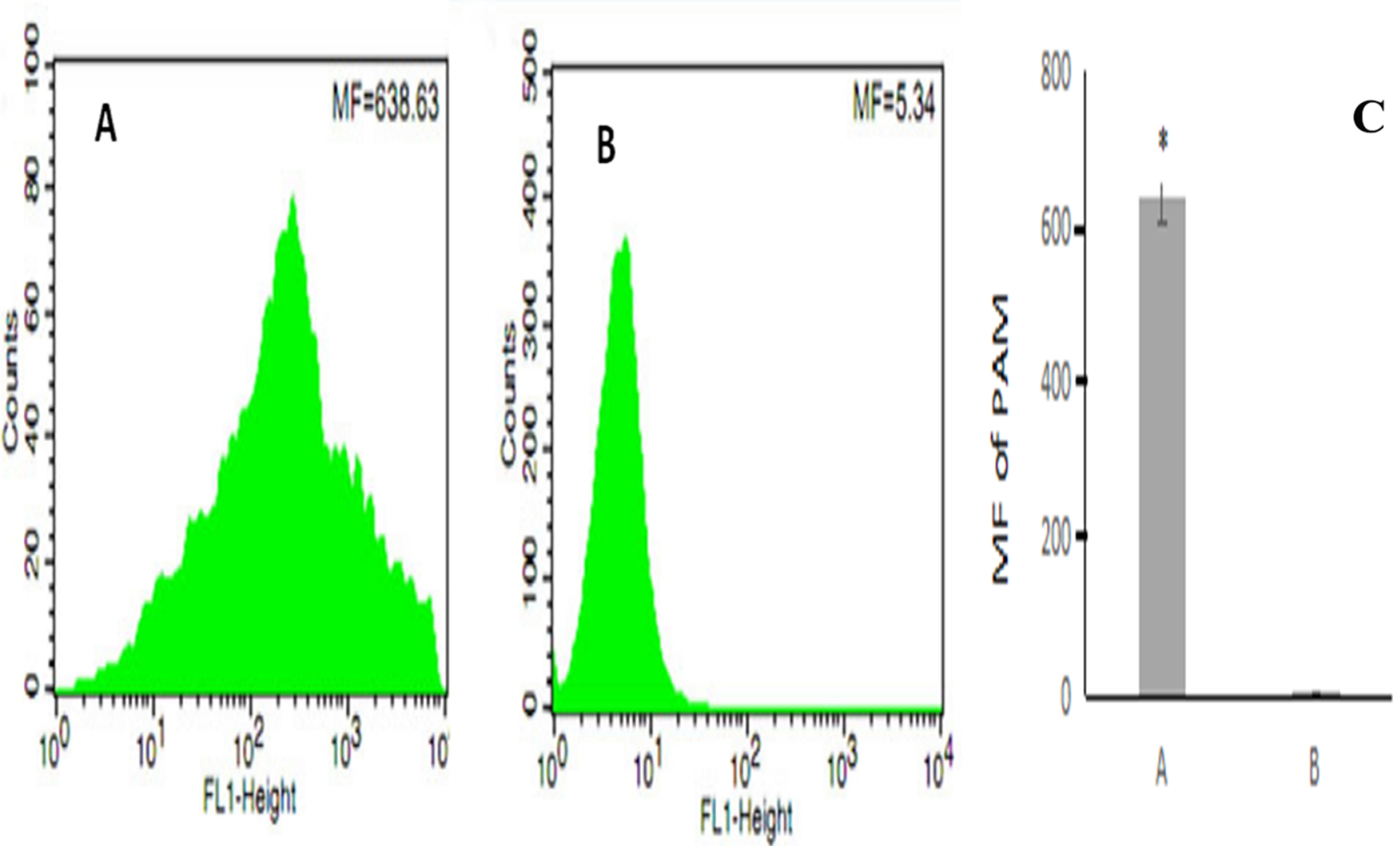
The flow cytometry analysis of PAM competive binding reaction. (A) showed that co-incubation group MF was 638.63; (B) showed the control MF was 5.34; and (C) showed the significantly difference between (A) and (B). (*, *P* < 0.01).

After erythrocyte lysis in the above co-incubation system, the samples were subjected to flow cytometry. The mean fluorescence intensity of PAMs in the co-incubation group was 638.63 ± 44.82 (Fig. 4A) whereas the mean fluorescence intensity of PAMs in the control group was 5.34 ± 0.08 (Fig. 4B); thus, the mean fluorescence intensity of PAMs in the co-incubation group was significantly higher than the control group (*P* < 0.01) (Fig. 4C).

### Level of CR1-like on porcine erythrocytes during the transfer of oposinized WT *E. coli*

**Figure 5:**
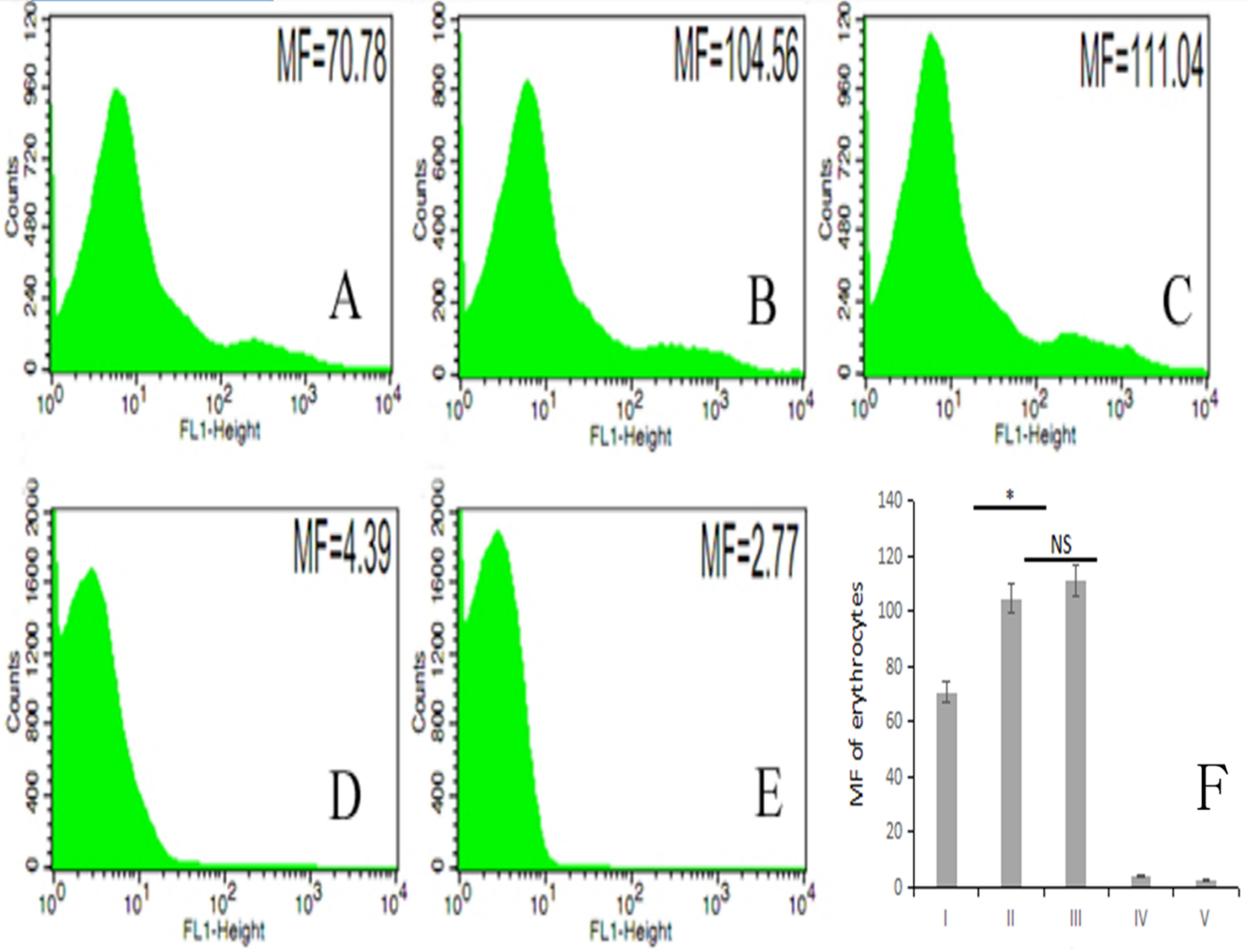
The MF of Group I, Group II, Group III, Group IV, and Group V. (A) 70.78; (B) 104.56; (C) 111.04; (D) 4.39; (E) 2.77; (F) the difference among the five groups (*P* < 0.05; *NS*, *P* > 0.05).

The fluorescence intensity of porcine erythrocytes as detected by flow cytometry for groups I–V was 70.78, 104.56, 111.04, 4.39, and 2.77, respectively (Figs. 5A–5E). The fluorescence intensities of porcine erythrocytes in groups I–III were significantly higher than those in groups IV and V (*P* < 0.05). The fluorescence intensity of porcine erythrocytes in group I was significantly lower than that of groups II and III (*P* < 0.05) whereas the fluorescence intensity of porcine erythrocytes in group II was lower than that in group III, but this difference was not statistically significant (*P* > 0.05) (Fig. 5F).

Thus, there were no significant changes in CR1-like expression levels of porcine erythrocytes between porcine erythrocytes alone and those co-incubated with PAMs. However, when porcine erythrocytes, fluorescent bacteria and PAMs were co-incubated, the expression levels of CR1-like on porcine erythrocytes decreased.

## Discussion

Alveolar macrophages (AM) provide an important defense line in the lungs for resisting pathogens. AMs participate in many physiological and pathological processes and play an important role in the body’s immune system (Rossow, 1998). Oposinized pathogens or ICs can activate various helper effector cells such as macrophages, neutrophils, and mast cells, and the Fc receptors on the surface of these cells can bind to the Fc region of ICs, thus activating the phagocytosis of these pathogens and completing the elimination of invading pathogens (Yang et al., 2014; Das, Biswas & Khera, 2013; Panda, Ravindran & Das, 2016). Erythrocytes clear ICs mainly via immune adhesion with the complement receptor (E-CR1) expressed on their cell membrane. When pathogens (such as viruses and bacteria), circulating ICs, and other molecules enter the bloodstream, they activate the complement pathway. The C3b fragment produced by C3 cleavage is coated on the surface of the pathogenic molecule to form an IC. CR1 specifically binds to C3b, thus adhering the IC on the erythrocyte surface. In addition, E-CR1 inhibits complement convertase (Yin et al., 2015, 2016) and factor I cofactor activity (Ross et al., 1982) and participates in immune regulation. In contrast, immune clearance functions of porcine erythrocytes and the molecular basis of erythrocyte-phagocyte interactions have not been reported. Therefore, we used the results of previous studies as a foundation to construct a co-incubation system of macrophages, erythrocytes, and fluorescent bacteria to investigate the interactions of two types of immune cells in the clearance of ICs in pigs.

Phagocytes use the receptors on their surface membrane to bind to bacteria, thus enabling macrophages to recognize invading pathogens non-specifically and enabling the body to activate the complement cascade quickly. This results in the production of large amounts of activated complement fragments, which bind covalently to pathogens. This promotes phagocytosis by macrophages, which have complement receptors (Taylor et al., 2005; Van Lookeren Campagne, Wiesmann & Brown, 2007; De Back et al., 2014). Our project group has previously proven that after GFP-*E. coli* oposinized by fresh serum activate complement fragments; the bacteria are coated with complement fragments and this causes immune adhesion reactions with porcine erythrocytes (Sun et al., 2012). We used laser confocal microscopy to directly observe the dynamic process of competitive binding of PAMs to oposinized WT *E. coli*. Porcine erythrocytes and PAMs both could bind to oposinized WT-*E. coli*-FITC and there were no significant changes in the adhesion status of fluorescent bacteria. As the observation time increased, the fluorescent intensity of these three cells weakened gradually and the fluorescence became clearer after focusing. However, the contours of porcine erythrocytes show a blurring phenomenon (Fig. 3). This shows that the weakening of the fluorescence intensity of the bacteria is not due to the quenching of fluorescence but is caused by the movement of fluorescent bacteria from their original adhesion location to the junction between erythrocytes and PAMs. At 2 h 59 min, no fluorescent bacteria were seen on the surface of porcine erythrocytes. Thus, PAMs can competitively bind to fluorescent bacteria that have adhered on the surface of porcine erythrocytes, and erythrocytes are not damaged during the phagocytosis of bacteria by PAMs. Flow cytometry results revealed that when PAMs are co-incubated with erythrocytes that were adhered with fluorescent bacteria, the fluorescent bacteria are transferred from porcine erythrocytes to the surface of PAMs; hence, positive signals could be detected by flow cytometry. Therefore, we can hypothesize that after erythrocytes have adhered to pathogens or ICs, they will pass through immune tissues such as those of the liver or spleen, during circulation. Resident immune cells in these tissues, such as macrophages, can competitively bind to pathogens and ICs on the surface of erythrocytes, thereby promoting clearance of complexes or antigens by cells of the innate immune system. Our study combines flow cytometry and laser confocal microscopy to directly describe this competitive binding phenomenon, which further clarifies this process and evidences the accuracy of these results.

Flow cytometry-based quantitation in our study revealed that interactions between porcine erythrocytes and PAMs alone will not cause changes in surface CR1-like, but the transfer of fluorescent bacteria from porcine erythrocytes to PAMs decreases CR1-like levels. Therefore, we hypothesize that during the inhibition of disease by the porcine immune system, exogenous pathogens can activate the immune adhesion functions of porcine erythrocytes by activating the complement system. During the interaction between porcine erythrocytes adhered to oposinized pathogens and PAMs, oposinized pathogens are transferred to PAMs, internalized, and eliminated. During this process, porcine erythrocytes are not damaged but the expression of CR1-like on its surface is decreased. Therefore, the immune function of porcine erythrocytes and the inhibition of diseases by the porcine immune system are in correlation. However, this needs to be verified by further studies; currently, our project group has researched related content.

## Conclusions

We used flow cytometry and laser confocal microscopy to obtain a preliminary examination of the interactions of porcine innate immune cells in the clearance of ICs. We found that PAMs can competitively bind to oposinized *E. coli* adhered to the surface of porcine erythrocytes. During this process, the physical morphology of porcine erythrocytes was not damaged, but the levels of its main functional protein CR1-like decreased. However, the exact molecular mechanism of competitive binding of bacteria between PAMs and erythrocytes and presentation of bacteria requires further study.

## Supplemental Information

10.7717/peerj.6439/supp-1 Supplemental Information 1 The raw data of the study. The Fig. 4 file contains the original flow cytometry data of the CR1-like quantity test results and Fig. 5 contains the original flow cytometry data of the E.coli transferation detection.

## Additional Information and Declarations

### Data Availability

The following information was supplied regarding data availability: The raw data are available in a Supplemental File.
