# Supplementary figures and images for: In vitro observation: the GFP-E. coli adhering to porcine erythrocytes can be removed by porcine alveolar macrophages
Source: PeerJ. 2019 Mar 8;7:e6439. doi: 10.7717/peerj.6439 (PMC6410693; doi:10.7717/peerj.6439)

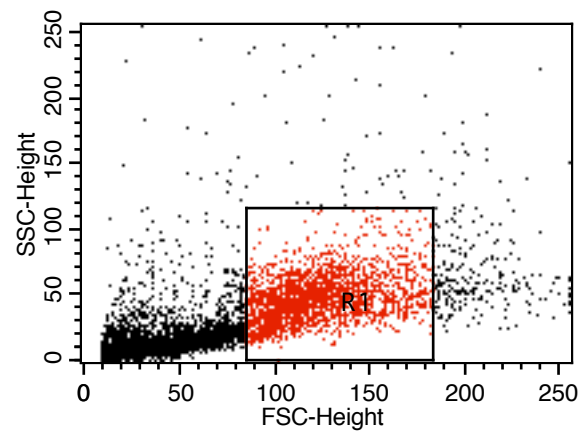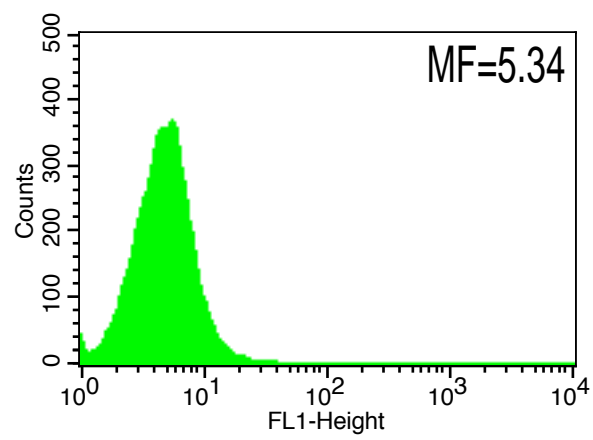

| Marker | Events | % Gated | Mean  | Median |
|--------|--------|---------|-------|--------|
| All    | 12044  | 100.00  | 5.34  | 4.70   |
| M1     | 109    | 0.91    | 22.40 | 20.54  |

Supplement: Supplemental Information 1 — The Fig. 4 file contains the original flow cytometry data of the CR1-like quantity test results and Fig. 5 contains the original flow cytometry data of the E.coli transferation detection. [file peerj-07-6439-s001.zip › Raw data/Fig. 4 raw data/PAM group/PAM Negative control group/PAM-1.pdf]

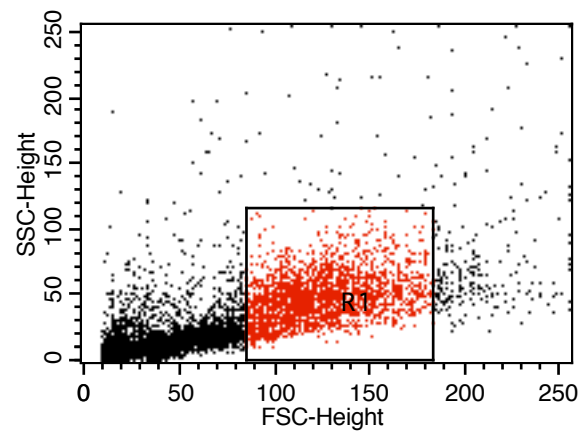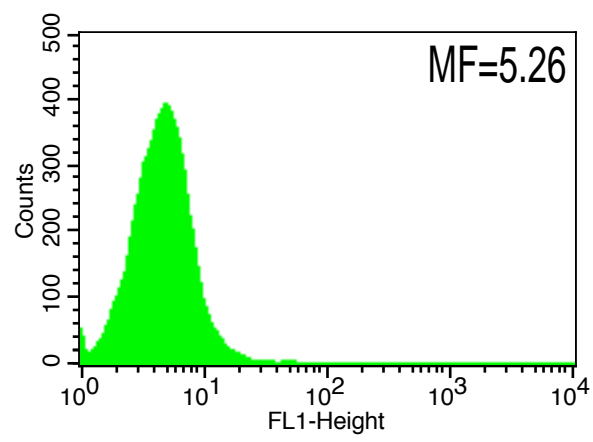

| Marker | Events | % Gated | Mean  | Median |
|--------|--------|---------|-------|--------|
| All    | 13162  | 100.00  | 5.26  | 4.70   |
| M1     | 146    | 1.11    | 23.98 | 20.54  |

Supplement: Supplemental Information 1 — The Fig. 4 file contains the original flow cytometry data of the CR1-like quantity test results and Fig. 5 contains the original flow cytometry data of the E.coli transferation detection. [file peerj-07-6439-s001.zip › Raw data/Fig. 4 raw data/PAM group/PAM Negative control group/PAM-2.pdf]

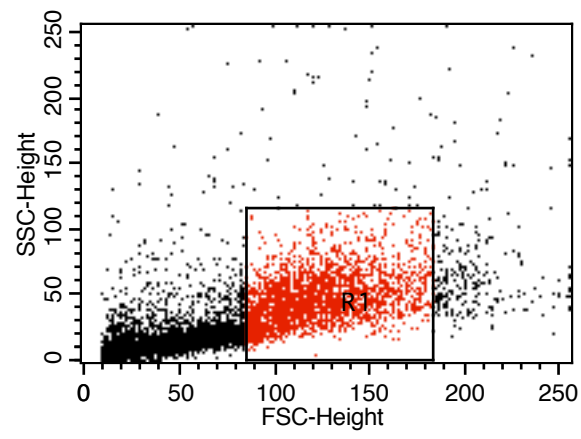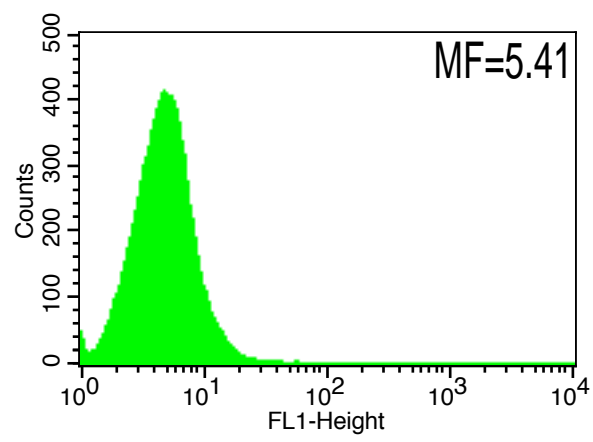

| Marker | Events | % Gated | Mean  | Median |
|--------|--------|---------|-------|--------|
| All    | 13985  | 100.00  | 5.41  | 4.70   |
| M1     | 147    | 1.05    | 25.24 | 20.54  |

Supplement: Supplemental Information 1 — The Fig. 4 file contains the original flow cytometry data of the CR1-like quantity test results and Fig. 5 contains the original flow cytometry data of the E.coli transferation detection. [file peerj-07-6439-s001.zip › Raw data/Fig. 4 raw data/PAM group/PAM Negative control group/PAM-3.pdf]

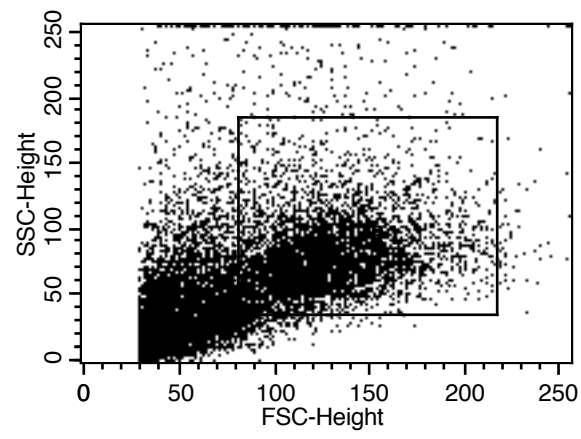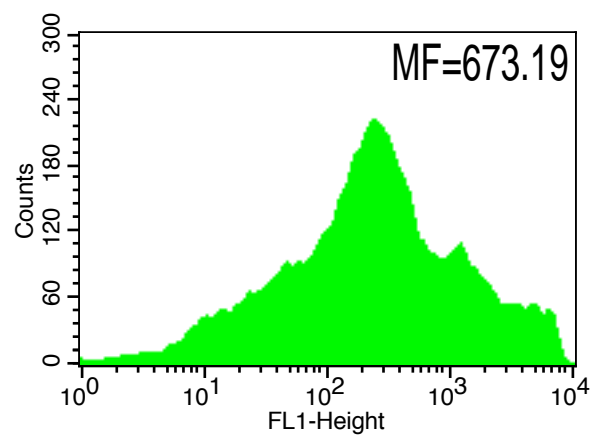

| Marker | Events | % Gated | Mean   | Median |
|--------|--------|---------|--------|--------|
| All    | 18475  | 100.00  | 673.19 | 228.76 |
| M1     | 17153  | 92.84   | 724.34 | 254.83 |

Supplement: Supplemental Information 1 — The Fig. 4 file contains the original flow cytometry data of the CR1-like quantity test results and Fig. 5 contains the original flow cytometry data of the E.coli transferation detection. [file peerj-07-6439-s001.zip › Raw data/Fig. 4 raw data/PAM group/PAM+FITC-wT/WT-E.coli-1.pdf]

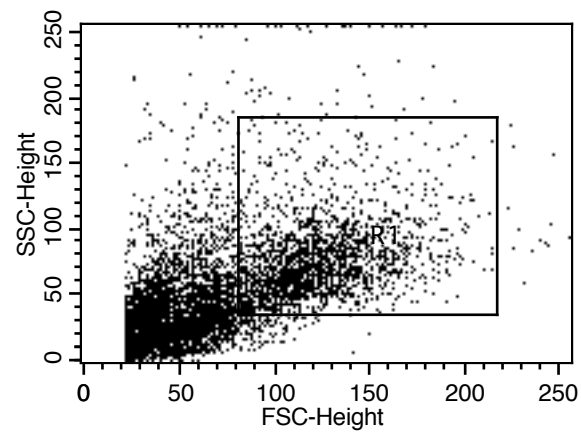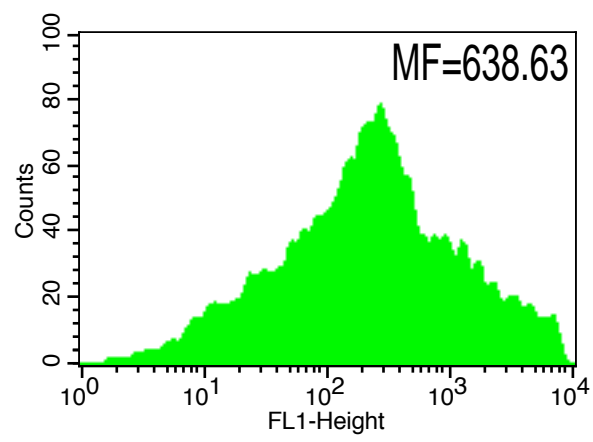

| Marker | Events | % Gated | Mean   | Median |
|--------|--------|---------|--------|--------|
| All    | 6710   | 100.00  | 638.63 | 212.88 |
| M1     | 6204   | 92.46   | 689.93 | 245.82 |

Supplement: Supplemental Information 1 — The Fig. 4 file contains the original flow cytometry data of the CR1-like quantity test results and Fig. 5 contains the original flow cytometry data of the E.coli transferation detection. [file peerj-07-6439-s001.zip › Raw data/Fig. 4 raw data/PAM group/PAM+FITC-wT/WT-E.coli-2.pdf]

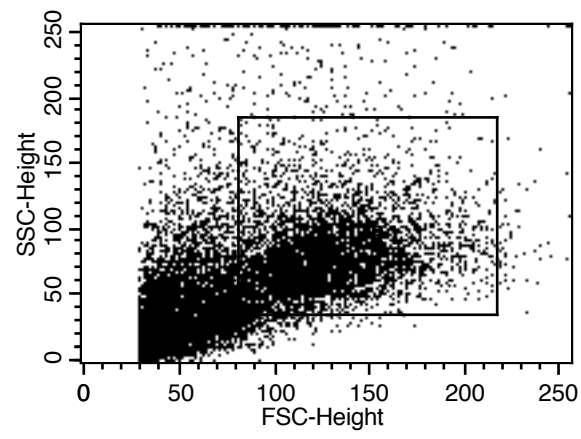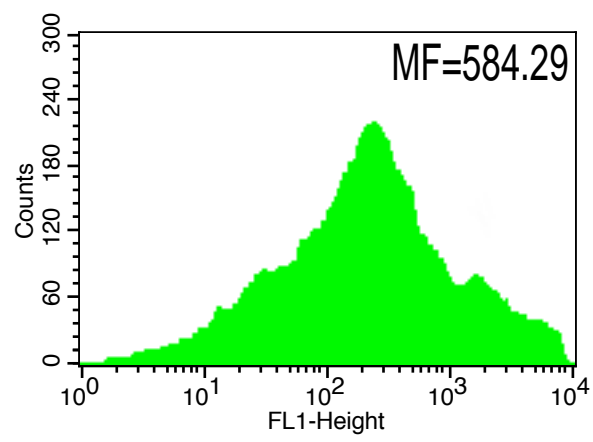

| Marker | Events | % Gated | Mean   | Median |
|--------|--------|---------|--------|--------|
| All    | 17258  | 100.00  | 584.29 | 198.54 |
| M1     | 15628  | 90.06   | 622.95 | 219.16 |

Supplement: Supplemental Information 1 — The Fig. 4 file contains the original flow cytometry data of the CR1-like quantity test results and Fig. 5 contains the original flow cytometry data of the E.coli transferation detection. [file peerj-07-6439-s001.zip › Raw data/Fig. 4 raw data/PAM group/PAM+FITC-wT/WT-E.coli-3.pdf]

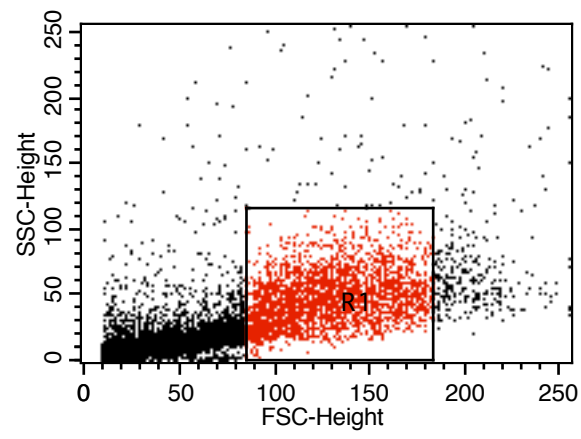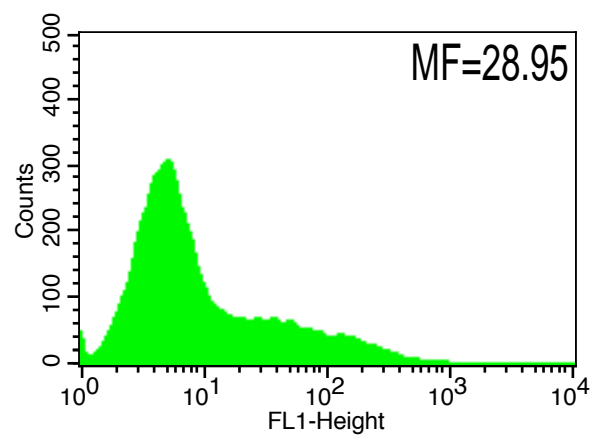

| Marker | Events | % Gated | Mean  | Median |
|--------|--------|---------|-------|--------|
| All    | 15166  | 100.00  | 28.95 | 6.26   |
| M1     | 4062   | 26.78   | 92.50 | 52.33  |

Supplement: Supplemental Information 1 — The Fig. 4 file contains the original flow cytometry data of the CR1-like quantity test results and Fig. 5 contains the original flow cytometry data of the E.coli transferation detection. [file peerj-07-6439-s001.zip › Raw data/Fig. 4 raw data/PAM group/PAM+GFP-E.coli/GFP-E.coli-1.pdf]

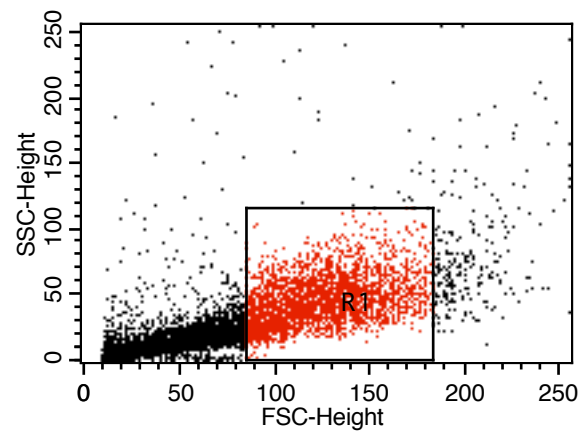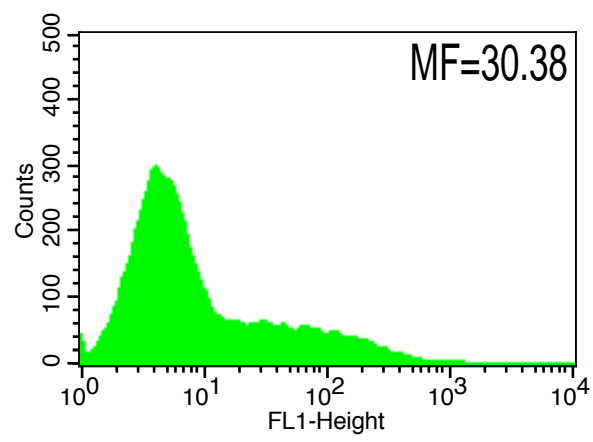

| Marker | Events | % Gated | Mean   | Median |
|--------|--------|---------|--------|--------|
| All    | 15176  | 100.00  | 30.38  | 6.04   |
| M1     | 3992   | 26.30   | 100.33 | 60.43  |

Supplement: Supplemental Information 1 — The Fig. 4 file contains the original flow cytometry data of the CR1-like quantity test results and Fig. 5 contains the original flow cytometry data of the E.coli transferation detection. [file peerj-07-6439-s001.zip › Raw data/Fig. 4 raw data/PAM group/PAM+GFP-E.coli/GFP-E.coli-2.pdf]

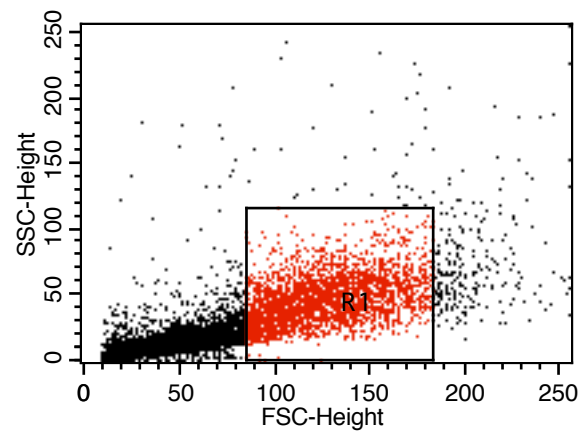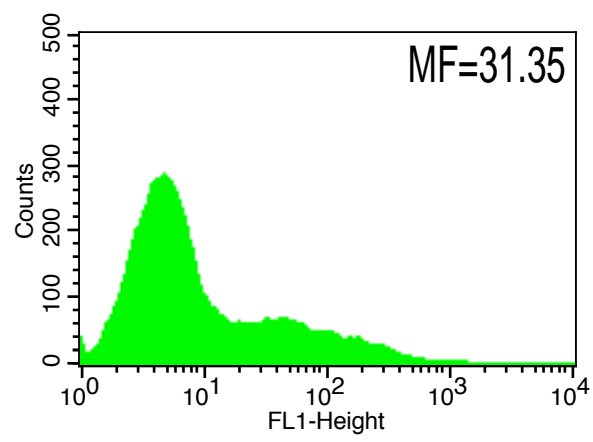

| Marker | Events | % Gated | Mean   | Median |
|--------|--------|---------|--------|--------|
| All    | 15197  | 100.00  | 31.35  | 6.04   |
| M1     | 4125   | 27.14   | 100.85 | 56.23  |

Supplement: Supplemental Information 1 — The Fig. 4 file contains the original flow cytometry data of the CR1-like quantity test results and Fig. 5 contains the original flow cytometry data of the E.coli transferation detection. [file peerj-07-6439-s001.zip › Raw data/Fig. 4 raw data/PAM group/PAM+GFP-E.coli/GFP-E.coli-3.pdf]

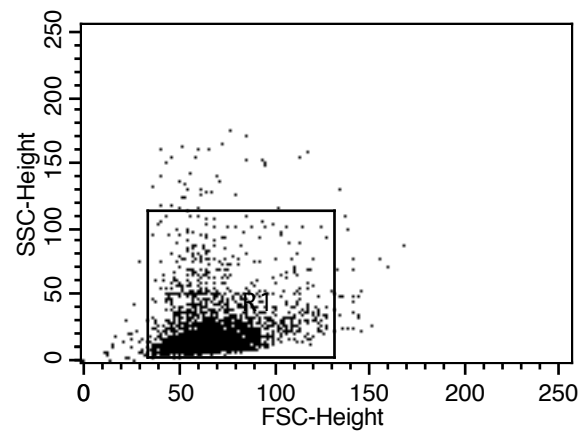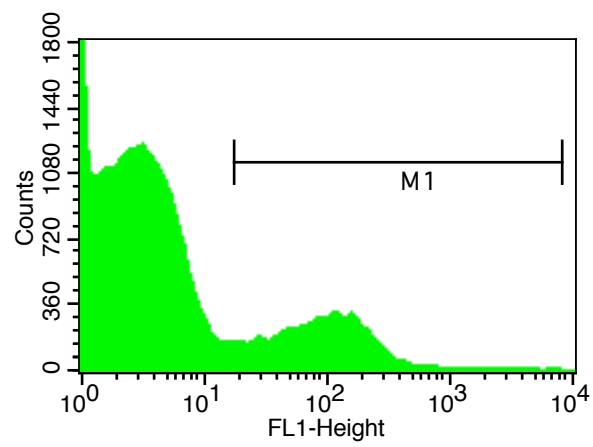

| Marker | Events | % Gated | Mean   |
|--------|--------|---------|--------|
| All    | 104309 | 100.00  | 27.47  |
| M1     | 18505  | 17.74   | 140.75 |

Supplement: Supplemental Information 1 — The Fig. 4 file contains the original flow cytometry data of the CR1-like quantity test results and Fig. 5 contains the original flow cytometry data of the E.coli transferation detection. [file peerj-07-6439-s001.zip › Raw data/Fig. 4 raw data/RBC group/║∞╧╕░√+GFP/║∞╧╕░√+GFP1.pdf]

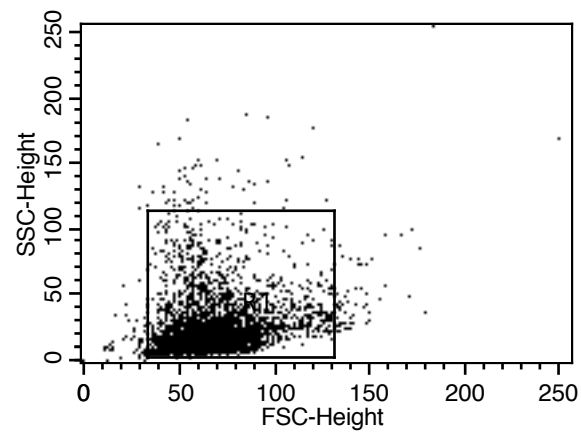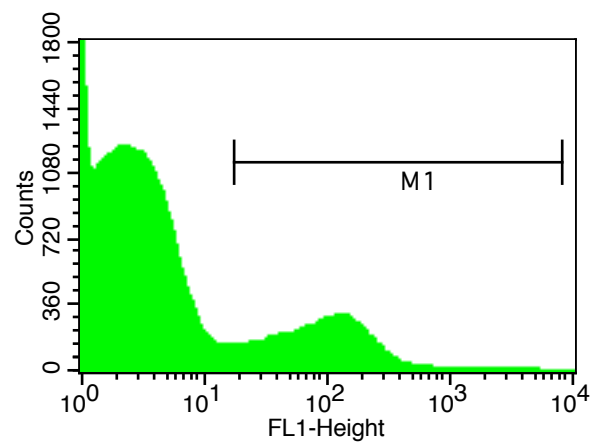

| Marker | Events | % Gated | Mean   |
|--------|--------|---------|--------|
| All    | 100931 | 100.00  | 25.85  |
| M1     | 17717  | 17.55   | 133.84 |

Supplement: Supplemental Information 1 — The Fig. 4 file contains the original flow cytometry data of the CR1-like quantity test results and Fig. 5 contains the original flow cytometry data of the E.coli transferation detection. [file peerj-07-6439-s001.zip › Raw data/Fig. 4 raw data/RBC group/║∞╧╕░√+GFP/║∞╧╕░√+GFP2.pdf]

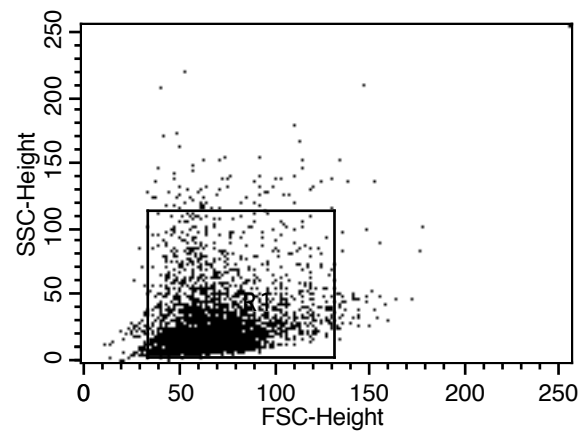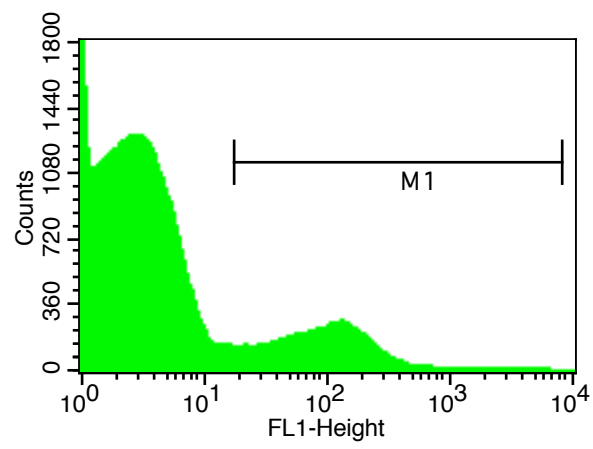

| Marker | Events | % Gated | Mean   |
|--------|--------|---------|--------|
| All    | 101242 | 100.00  | 24.72  |
| M1     | 15439  | 15.25   | 145.83 |

Supplement: Supplemental Information 1 — The Fig. 4 file contains the original flow cytometry data of the CR1-like quantity test results and Fig. 5 contains the original flow cytometry data of the E.coli transferation detection. [file peerj-07-6439-s001.zip › Raw data/Fig. 4 raw data/RBC group/║∞╧╕░√+GFP/║∞╧╕░√+GFP3.pdf]

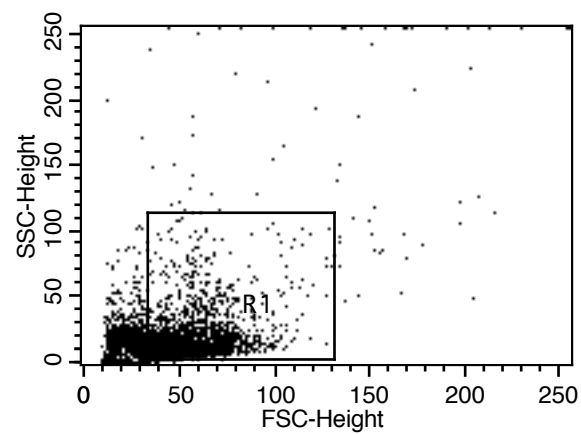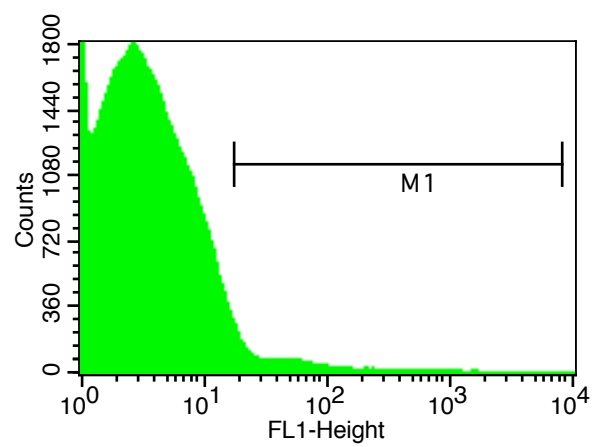

| Marker | Events | % Gated | Mean  |
|--------|--------|---------|-------|
| All    | 118154 | 100.00  | 6.20  |
| M1     | 4470   | 3.78    | 65.02 |

Supplement: Supplemental Information 1 — The Fig. 4 file contains the original flow cytometry data of the CR1-like quantity test results and Fig. 5 contains the original flow cytometry data of the E.coli transferation detection. [file peerj-07-6439-s001.zip › Raw data/Fig. 4 raw data/RBC group/║∞╧╕░√+GFP+PAM/║∞╧╕░√+GFP+PAM1.pdf]

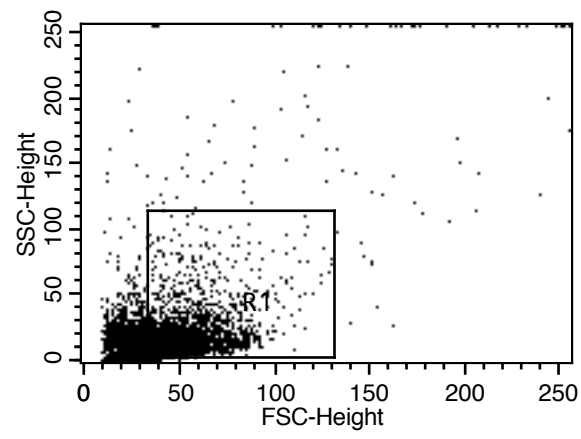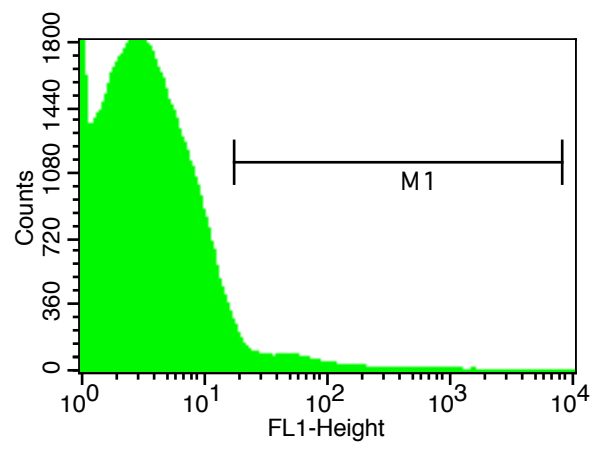

| Marker | Events | % Gated | Mean  |
|--------|--------|---------|-------|
| All    | 123474 | 100.00  | 6.44  |
| M1     | 5028   | 4.07    | 67.65 |

Supplement: Supplemental Information 1 — The Fig. 4 file contains the original flow cytometry data of the CR1-like quantity test results and Fig. 5 contains the original flow cytometry data of the E.coli transferation detection. [file peerj-07-6439-s001.zip › Raw data/Fig. 4 raw data/RBC group/║∞╧╕░√+GFP+PAM/║∞╧╕░√+GFP+PAM2.pdf]

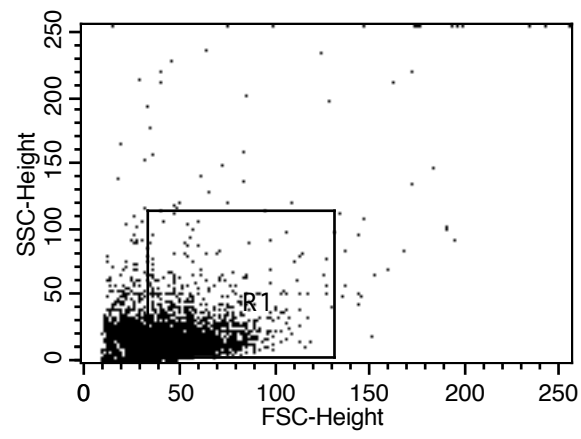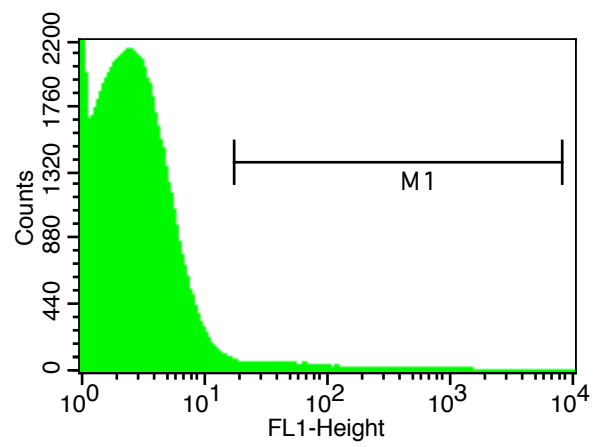

| Marker | Events | % Gated | Mean  |
|--------|--------|---------|-------|
| All    | 121498 | 100.00  | 4.30  |
| M1     | 2351   | 1.94    | 79.39 |

Supplement: Supplemental Information 1 — The Fig. 4 file contains the original flow cytometry data of the CR1-like quantity test results and Fig. 5 contains the original flow cytometry data of the E.coli transferation detection. [file peerj-07-6439-s001.zip › Raw data/Fig. 4 raw data/RBC group/║∞╧╕░√+GFP+PAM/║∞╧╕░√+GFP+PAM3.pdf]

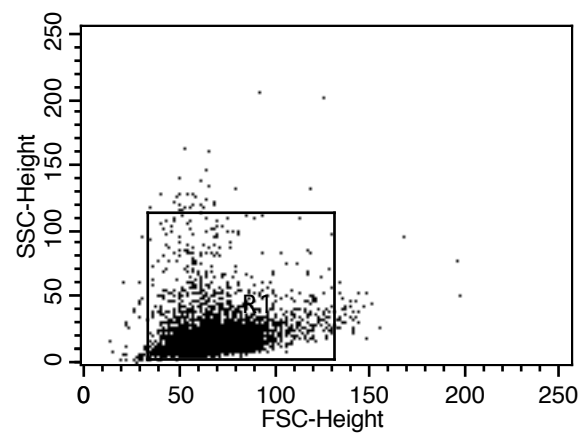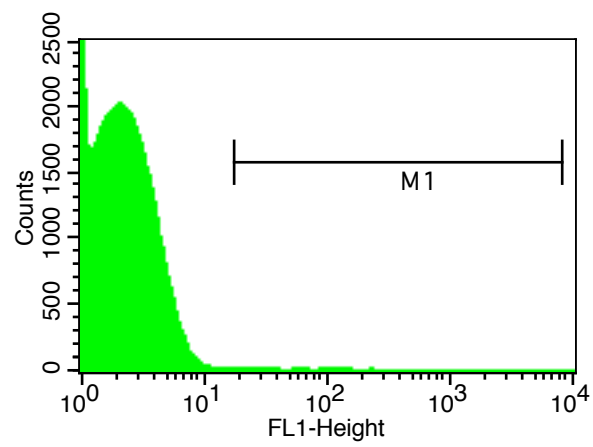

| Marker | Events | % Gated | Mean   |
|--------|--------|---------|--------|
| All    | 100715 | 100.00  | 2.35   |
| M1     | 67     | 0.07    | 114.72 |

Supplement: Supplemental Information 1 — The Fig. 4 file contains the original flow cytometry data of the CR1-like quantity test results and Fig. 5 contains the original flow cytometry data of the E.coli transferation detection. [file peerj-07-6439-s001.zip › Raw data/Fig. 4 raw data/RBC group/║∞╧╕░√╥⌡╨╘╢╘╒╒╫Θ/║∞╧╕░√╢╘╒╒╫Θ1.pdf]

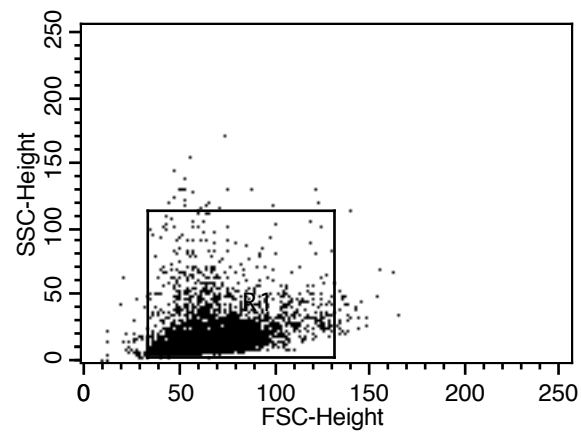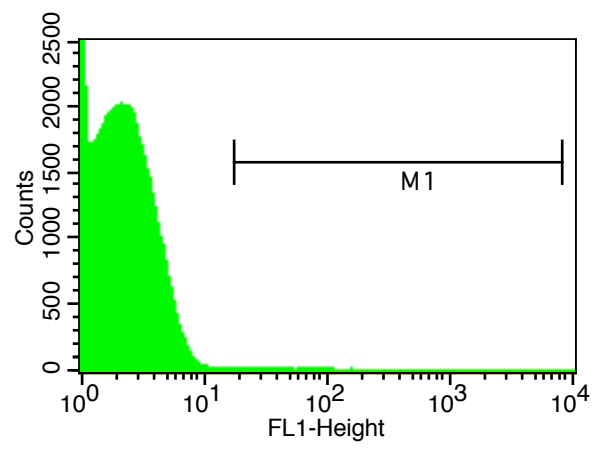

| Marker | Events | % Gated | Mean   |
|--------|--------|---------|--------|
| All    | 100775 | 100.00  | 2.36   |
| M1     | 73     | 0.07    | 159.79 |

Supplement: Supplemental Information 1 — The Fig. 4 file contains the original flow cytometry data of the CR1-like quantity test results and Fig. 5 contains the original flow cytometry data of the E.coli transferation detection. [file peerj-07-6439-s001.zip › Raw data/Fig. 4 raw data/RBC group/║∞╧╕░√╥⌡╨╘╢╘╒╒╫Θ/║∞╧╕░√╢╘╒╒╫Θ2.pdf]

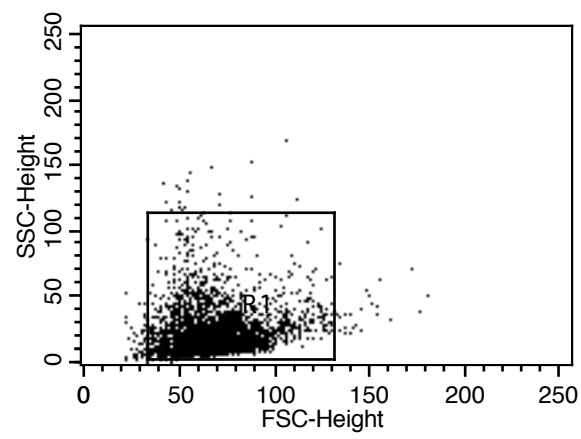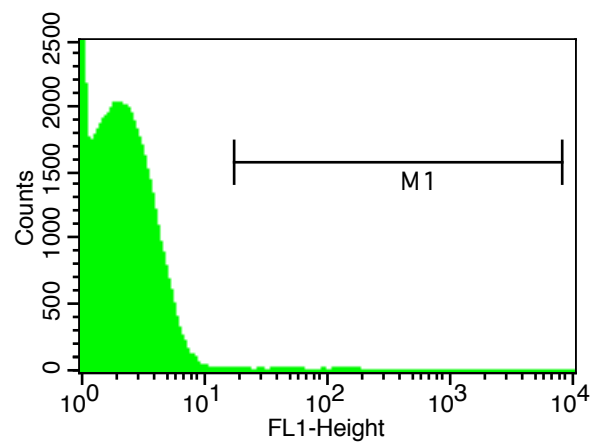

| Marker | Events | % Gated | Mean  |
|--------|--------|---------|-------|
| All    | 100958 | 100.00  | 2.28  |
| M1     | 56     | 0.06    | 89.73 |

Supplement: Supplemental Information 1 — The Fig. 4 file contains the original flow cytometry data of the CR1-like quantity test results and Fig. 5 contains the original flow cytometry data of the E.coli transferation detection. [file peerj-07-6439-s001.zip › Raw data/Fig. 4 raw data/RBC group/║∞╧╕░√╥⌡╨╘╢╘╒╒╫Θ/║∞╧╕░√╢╘╒╒╫Θ3.pdf]

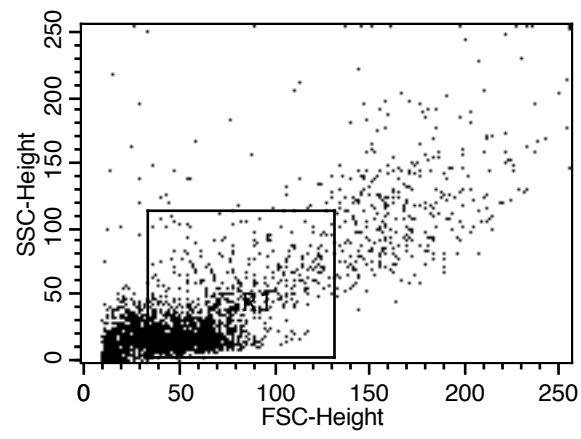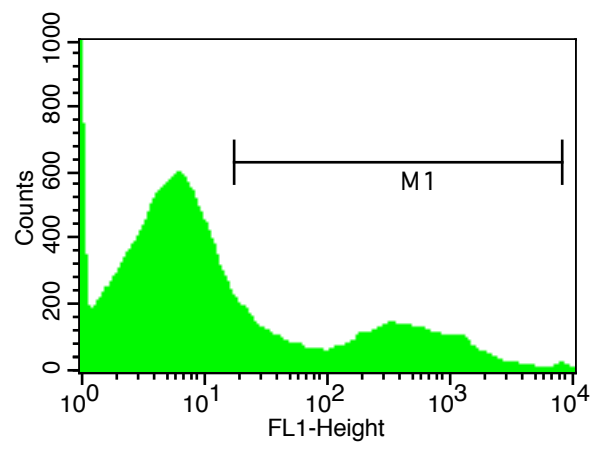

| Marker | Events | % Gated | Mean   | Median |
|--------|--------|---------|--------|--------|
| All    | 46551  | 100.00  | 163.43 | 7.50   |
| M1     | 14292  | 30.70   | 515.13 | 220.67 |

Supplement: Supplemental Information 1 — The Fig. 4 file contains the original flow cytometry data of the CR1-like quantity test results and Fig. 5 contains the original flow cytometry data of the E.coli transferation detection. [file peerj-07-6439-s001.zip › Raw data/Fig. 5 raw data/ó± group/PAM-1.pdf]

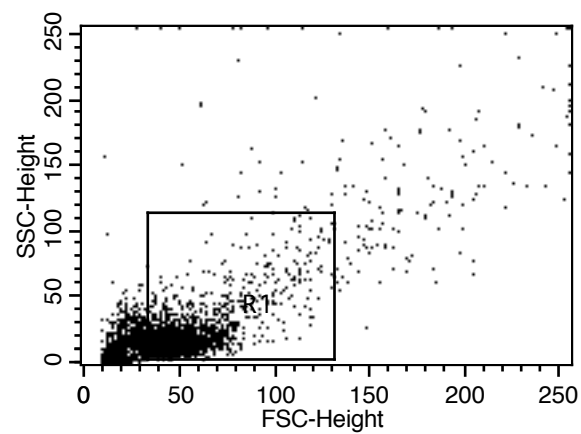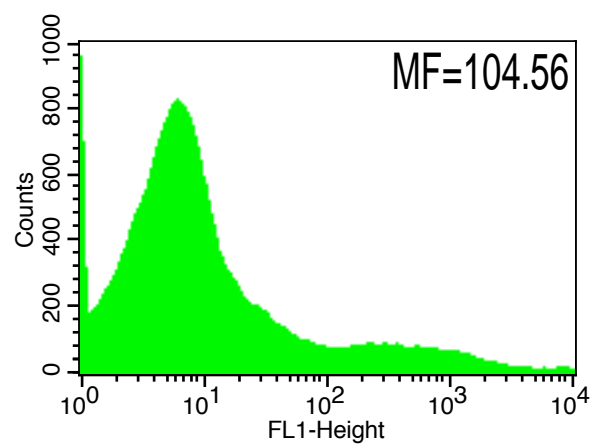

| Marker | Events | % Gated | Mean   | Median |
|--------|--------|---------|--------|--------|
| All    | 52371  | 100.00  | 104.56 | 6.98   |
| M1     | 13205  | 25.21   | 390.61 | 74.99  |

Supplement: Supplemental Information 1 — The Fig. 4 file contains the original flow cytometry data of the CR1-like quantity test results and Fig. 5 contains the original flow cytometry data of the E.coli transferation detection. [file peerj-07-6439-s001.zip › Raw data/Fig. 5 raw data/ó± group/PAM-2.pdf]

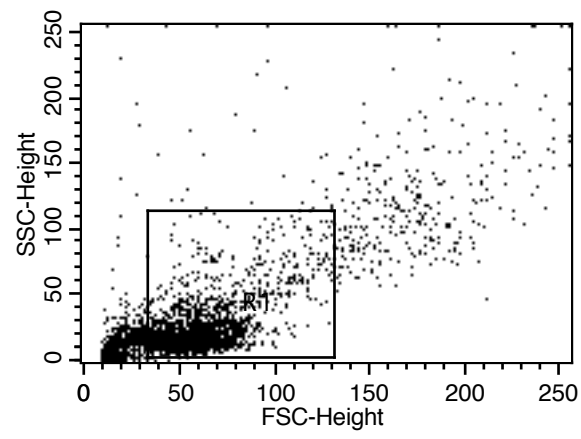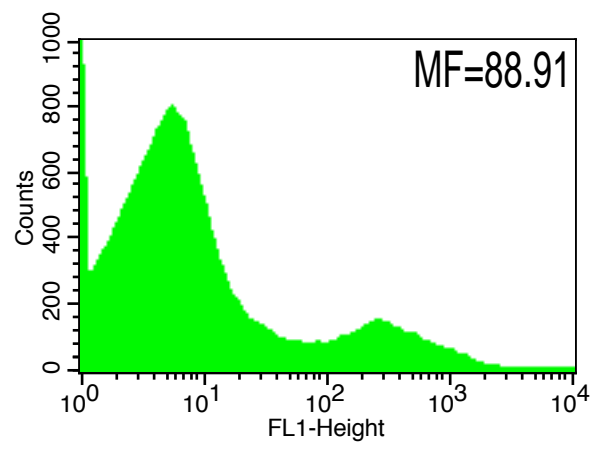

| Marker | Events | % Gated | Mean   | Median |
|--------|--------|---------|--------|--------|
| All    | 51734  | 100.00  | 88.91  | 6.57   |
| M1     | 11277  | 21.79   | 353.74 | 162.33 |

Supplement: Supplemental Information 1 — The Fig. 4 file contains the original flow cytometry data of the CR1-like quantity test results and Fig. 5 contains the original flow cytometry data of the E.coli transferation detection. [file peerj-07-6439-s001.zip › Raw data/Fig. 5 raw data/ó± group/PAM-3.pdf]

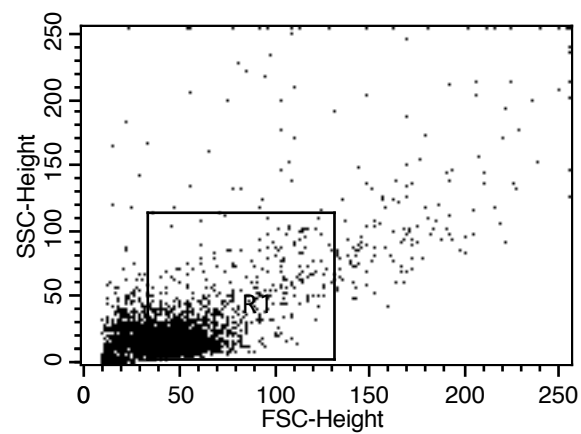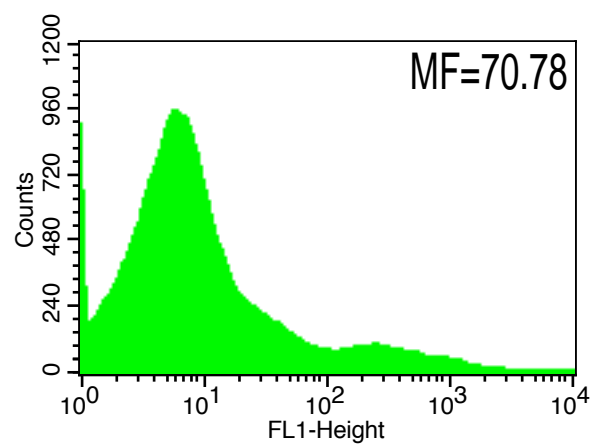

| Marker | Events | % Gated | Mean   | Median |
|--------|--------|---------|--------|--------|
| All    | 58924  | 100.00  | 70.78  | 6.98   |
| M1     | 14041  | 23.83   | 265.04 | 58.29  |

Supplement: Supplemental Information 1 — The Fig. 4 file contains the original flow cytometry data of the CR1-like quantity test results and Fig. 5 contains the original flow cytometry data of the E.coli transferation detection. [file peerj-07-6439-s001.zip › Raw data/Fig. 5 raw data/ó≥ group/Transfer group-1.pdf]

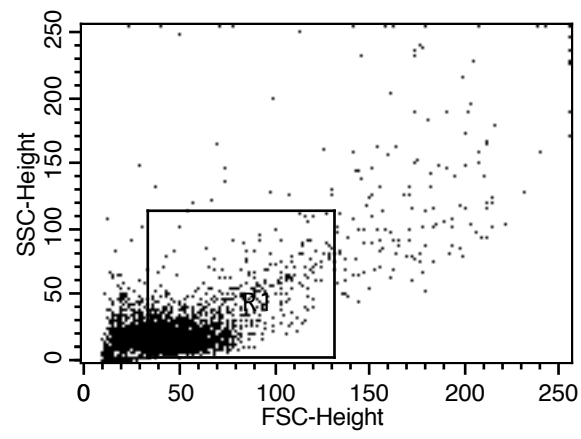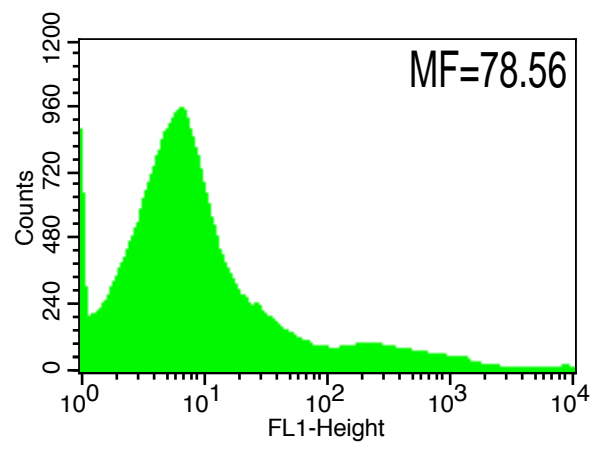

| Marker | Events | % Gated | Mean   | Median |
|--------|--------|---------|--------|--------|
| All    | 52738  | 100.00  | 78.56  | 7.53   |
| M1     | 11365  | 21.56   | 289.32 | 63.21  |

Supplement: Supplemental Information 1 — The Fig. 4 file contains the original flow cytometry data of the CR1-like quantity test results and Fig. 5 contains the original flow cytometry data of the E.coli transferation detection. [file peerj-07-6439-s001.zip › Raw data/Fig. 5 raw data/ó≥ group/Transfer group-2.pdf]

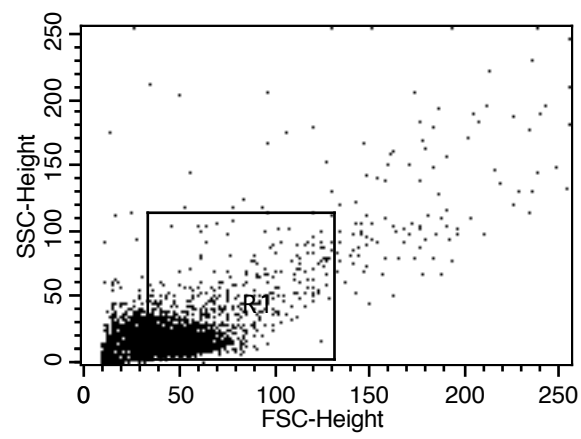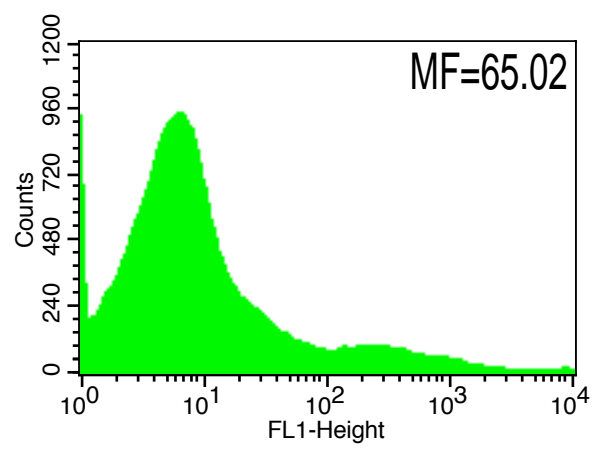

| Marker | Events | % Gated | Mean   | Median |
|--------|--------|---------|--------|--------|
| All    | 59621  | 100.00  | 65.02  | 6.07   |
| M1     | 12863  | 21.57   | 252.37 | 56.33  |

Supplement: Supplemental Information 1 — The Fig. 4 file contains the original flow cytometry data of the CR1-like quantity test results and Fig. 5 contains the original flow cytometry data of the E.coli transferation detection. [file peerj-07-6439-s001.zip › Raw data/Fig. 5 raw data/ó≥ group/Transfer group-3.pdf]

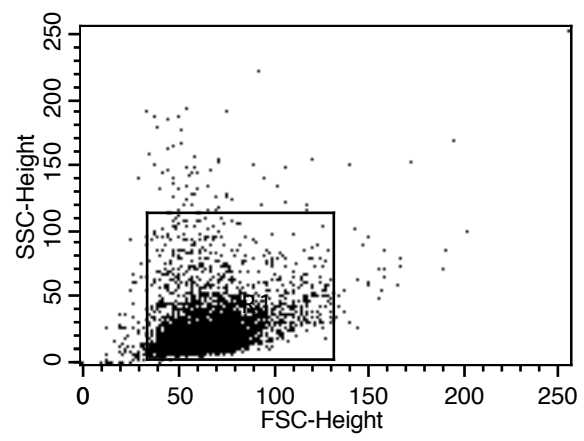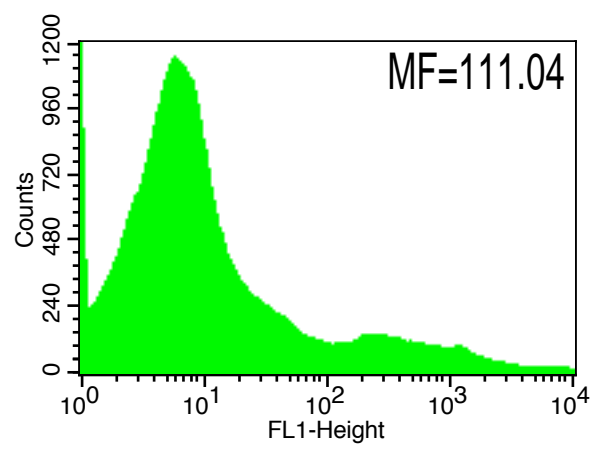

| Marker | Events | % Gated | Mean   | Median |
|--------|--------|---------|--------|--------|
| All    | 73278  | 100.00  | 111.04 | 6.98   |
| M1     | 19088  | 26.05   | 394.53 | 80.58  |

Supplement: Supplemental Information 1 — The Fig. 4 file contains the original flow cytometry data of the CR1-like quantity test results and Fig. 5 contains the original flow cytometry data of the E.coli transferation detection. [file peerj-07-6439-s001.zip › Raw data/Fig. 5 raw data/ó≤ group/Control-1.pdf]

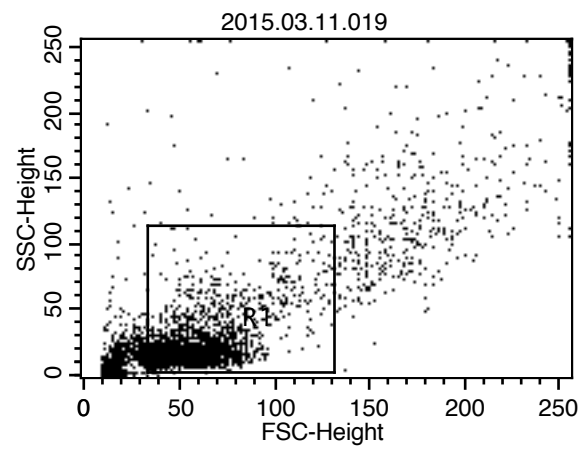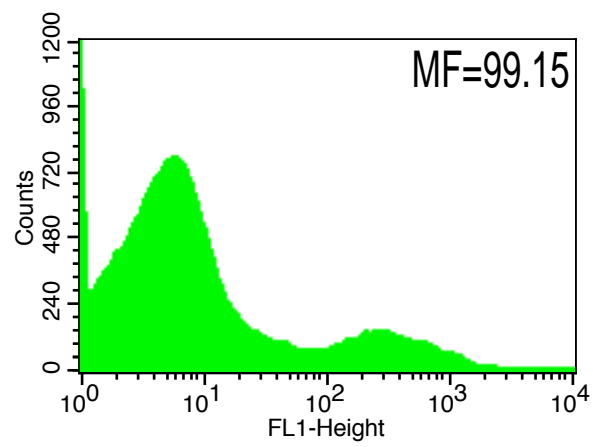

| Marker | Events | % Gated | Mean   | Median |
|--------|--------|---------|--------|--------|
| All    | 50573  | 100.00  | 99.15  | 6.23   |
| M1     | 11237  | 22.18   | 383.14 | 176.51 |

Supplement: Supplemental Information 1 — The Fig. 4 file contains the original flow cytometry data of the CR1-like quantity test results and Fig. 5 contains the original flow cytometry data of the E.coli transferation detection. [file peerj-07-6439-s001.zip › Raw data/Fig. 5 raw data/ó≤ group/Control-2.pdf]

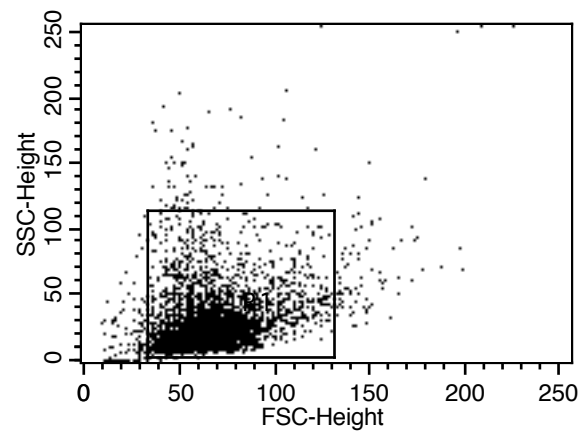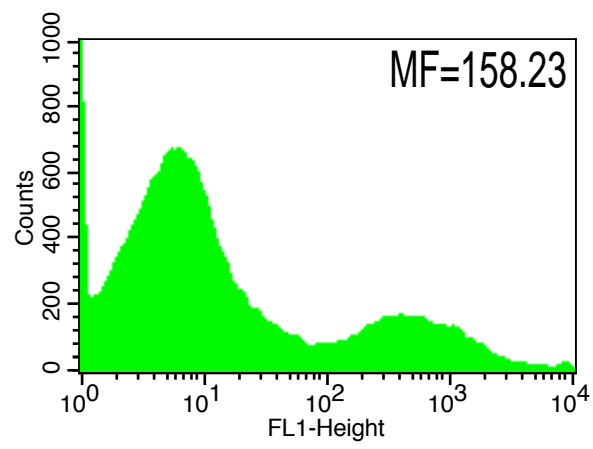

| Marker | Events | % Gated | Mean   | Median |
|--------|--------|---------|--------|--------|
| All    | 57833  | 100.00  | 158.23 | 7.16   |
| M1     | 18536  | 32.02   | 452.53 | 194.47 |

Supplement: Supplemental Information 1 — The Fig. 4 file contains the original flow cytometry data of the CR1-like quantity test results and Fig. 5 contains the original flow cytometry data of the E.coli transferation detection. [file peerj-07-6439-s001.zip › Raw data/Fig. 5 raw data/ó≤ group/Control-3.pdf]

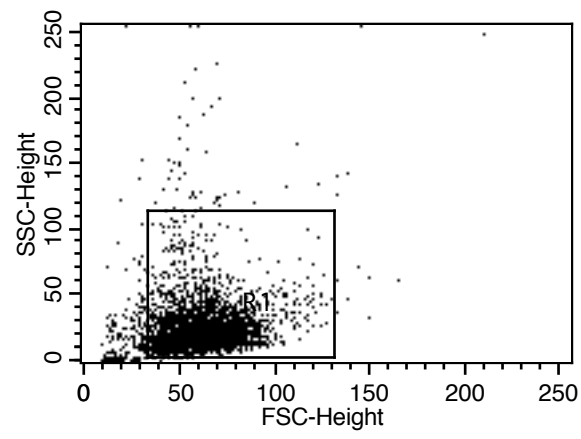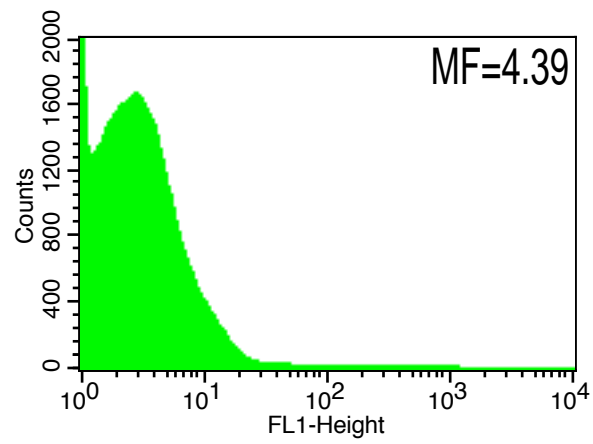

| Marker | Events | % Gated | Mean  | Median |
|--------|--------|---------|-------|--------|
| All    | 102069 | 100.00  | 4.39  | 2.37   |
| M1     | 1745   | 1.71    | 71.88 | 25.48  |

Supplement: Supplemental Information 1 — The Fig. 4 file contains the original flow cytometry data of the CR1-like quantity test results and Fig. 5 contains the original flow cytometry data of the E.coli transferation detection. [file peerj-07-6439-s001.zip › Raw data/Fig. 5 raw data/ó⌠ group/Flow cytometry isotype control-1.pdf]

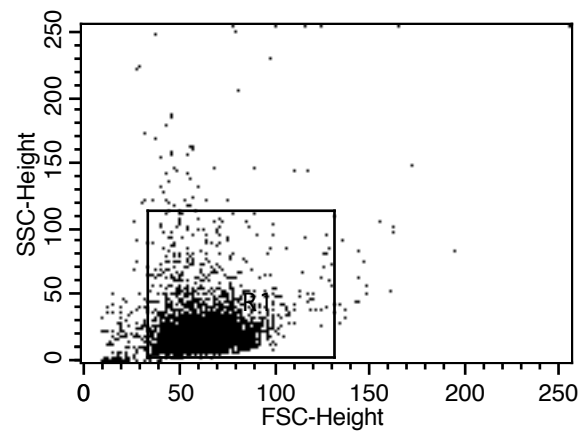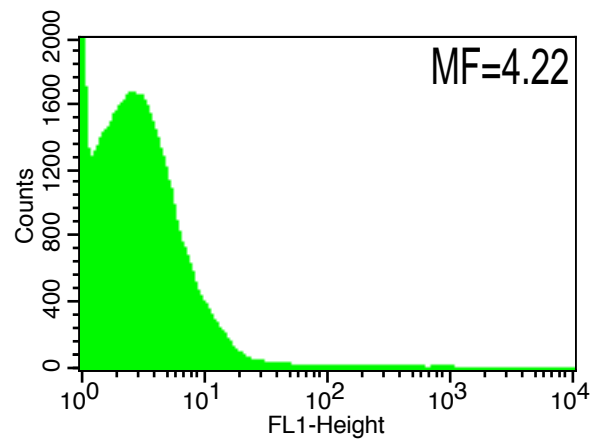

| Marker | Events | % Gated | Mean  | Median |
|--------|--------|---------|-------|--------|
| All    | 101706 | 100.00  | 4.22  | 2.37   |
| M1     | 1711   | 1.68    | 64.11 | 25.48  |

Supplement: Supplemental Information 1 — The Fig. 4 file contains the original flow cytometry data of the CR1-like quantity test results and Fig. 5 contains the original flow cytometry data of the E.coli transferation detection. [file peerj-07-6439-s001.zip › Raw data/Fig. 5 raw data/ó⌠ group/Flow cytometry isotype control-2.pdf]

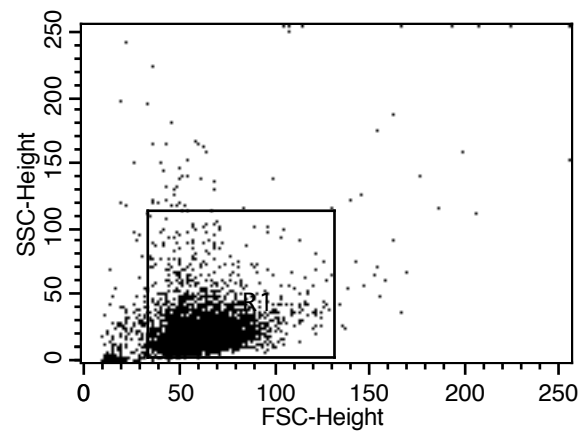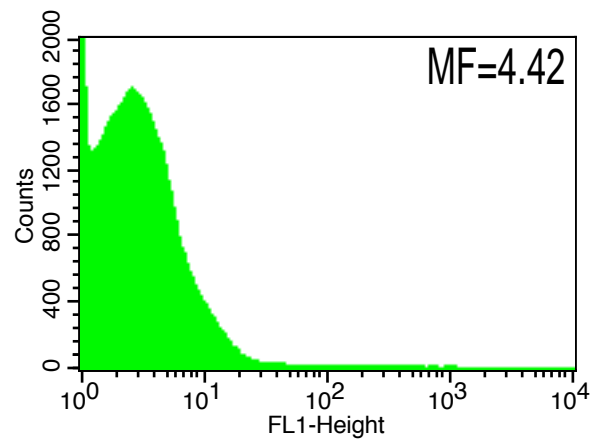

| Marker | Events | % Gated | Mean  | Median |
|--------|--------|---------|-------|--------|
| All    | 101792 | 100.00  | 4.42  | 2.37   |
| M1     | 1740   | 1.71    | 76.24 | 25.48  |

Supplement: Supplemental Information 1 — The Fig. 4 file contains the original flow cytometry data of the CR1-like quantity test results and Fig. 5 contains the original flow cytometry data of the E.coli transferation detection. [file peerj-07-6439-s001.zip › Raw data/Fig. 5 raw data/ó⌠ group/Flow cytometry isotype control-3.pdf]

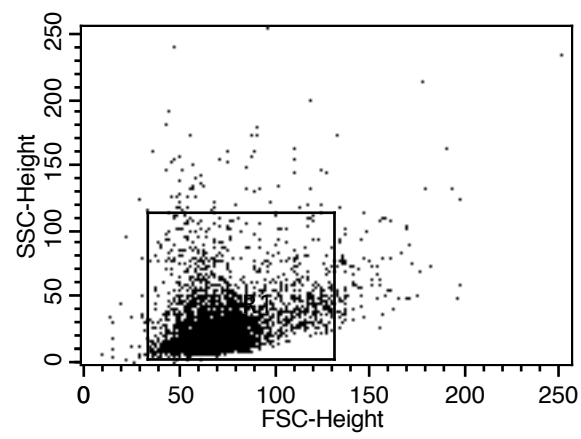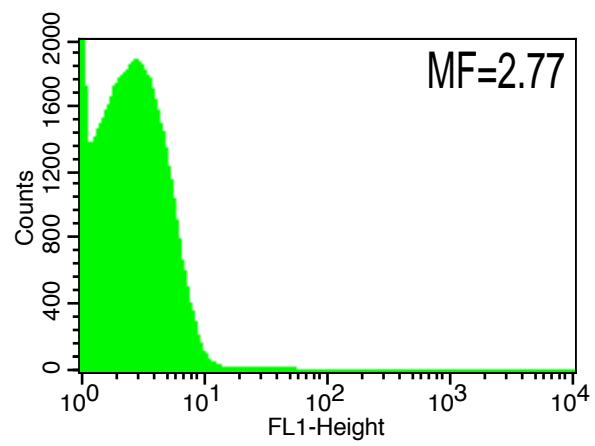

| Marker | Events | % Gated | Mean  | Median |
|--------|--------|---------|-------|--------|
| All    | 100103 | 100.00  | 2.77  | 2.21   |
| M1     | 56     | 0.06    | 33.41 | 22.88  |

Supplement: Supplemental Information 1 — The Fig. 4 file contains the original flow cytometry data of the CR1-like quantity test results and Fig. 5 contains the original flow cytometry data of the E.coli transferation detection. [file peerj-07-6439-s001.zip › Raw data/Fig. 5 raw data/ó⌡ group/RBC1.pdf]

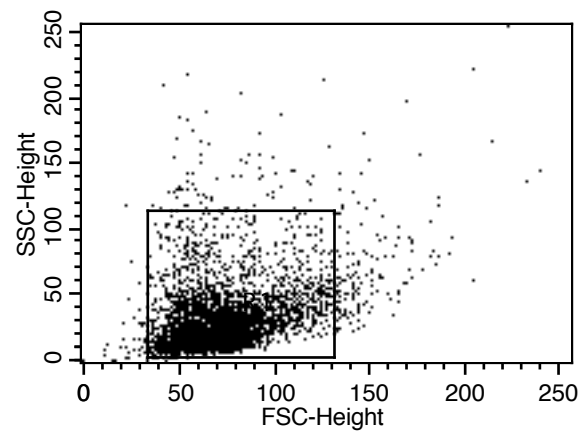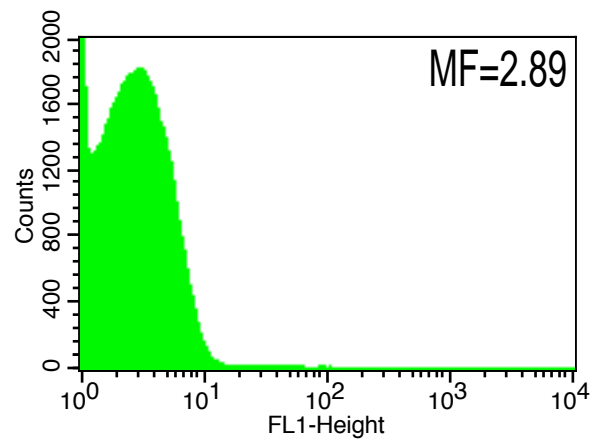

| Marker | Events | % Gated | Mean  | Median |
|--------|--------|---------|-------|--------|
| All    | 100334 | 100.00  | 2.89  | 2.29   |
| M1     | 87     | 0.09    | 52.14 | 25.48  |

Supplement: Supplemental Information 1 — The Fig. 4 file contains the original flow cytometry data of the CR1-like quantity test results and Fig. 5 contains the original flow cytometry data of the E.coli transferation detection. [file peerj-07-6439-s001.zip › Raw data/Fig. 5 raw data/ó⌡ group/RBC2.pdf]

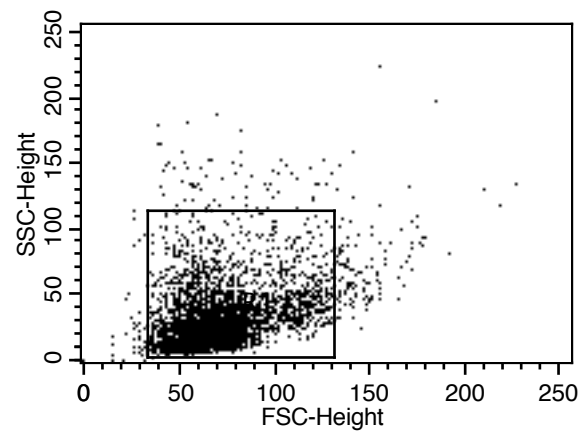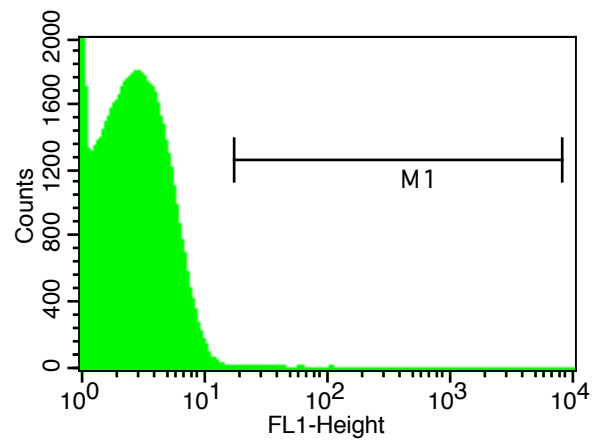

| Marker | Events | % Gated | Mean  | Median |
|--------|--------|---------|-------|--------|
| All    | 100582 | 100.00  | 2.86  | 2.29   |
| M1     | 78     | 0.08    | 43.90 | 25.03  |

Supplement: Supplemental Information 1 — The Fig. 4 file contains the original flow cytometry data of the CR1-like quantity test results and Fig. 5 contains the original flow cytometry data of the E.coli transferation detection. [file peerj-07-6439-s001.zip › Raw data/Fig. 5 raw data/ó⌡ group/RBC3.pdf]
